# Genetic Determinants of Circulating Adiponectin Levels, a Complex Multifactorial Intermediate Phenotype

**DOI:** 10.3390/biom16101474

**Published:** 2026-10-10

**Authors:** Katalin Csép

**Affiliations:** Genetics Department, George Emil Palade University of Medicine, Pharmacy, Science and Technology of Târgu Mureş, 540142 Târgu Mureş, Romania; katalin.csep@umfst.ro

**Keywords:** adiponectin, ADIPOQ, adiponectin receptor, ADIPOR, T-cadherin, CDH13, heritability, genetic variation, GWAS, linkage

## Abstract

Circulating adiponectin represents a complex trait with a multifactorial background influenced by internal and external genetic and non-genetic factors, as well as physiological and pathological conditions. It is an important intermediate phenotype characterized by unusually high levels, paradoxical associations with obesity or mortality, and analytical difficulties. Family studies, genome-wide linkage and association analyses demonstrate genetics as a major determinant, though possibly of lesser importance than in similar traits. Heritability values estimated by classical family studies vary significantly (30–93%), depending on study design, age, sex and ancestry. The strongest signals characterized by population differences are on chromosome 3 and 16, where *ADIPOQ* and *CDH13* encoding adiponectin and T-cadherin reside, but tens of other loci situated on almost all chromosomes and genetic variants related to over 150 genes are suspected to modulate plasma levels, frequently by unexplained mechanisms. Population-specific high-impact rare missense mutations (e.g., G45R, R55C, G84R, G90S, R112C, I164T) and common synonymous exon (rs2241766), intron (e.g., rs1501299, rs17366568) or regulatory sequence (e.g., rs17300539, rs266729, rs6773957) variants of *ADIPOQ*, often in linkage disequilibrium, affect circulating adiponectin levels, usually by altering multimer assembly or gene expression regulation. Three decades of adiponectin genetics research contributed significantly to deciphering adiponectin biology, and emerging genomic technologies may clarify current knowledge gaps.

## 1. Introduction

Adiponectin is a key homeostatic factor, a unique hormone and the most abundant adipokine, sometimes called a fire-fighter or guardian angel adipocytokine due to its insulin-sensitizing, anti-inflammatory, anti-oxidant, anti-fibrotic, anti-apoptotic functions, and protective effects against various diseases such as type 2 diabetes, atherosclerosis, liver steatosis or neoplasia [1]. As an adipocyte-derived cell signaling molecule, it exerts endocrine, paracrine and autocrine effects. Carried by the bloodstream to distant target cells, adiponectin mediates the crosstalk between the adipose tissue and metabolic organs, orchestrating numerous biological processes critical for whole body homeostasis [2]. Plasma adiponectin represents an intermediate phenotype of major importance for health and disease, a useful biomarker in various conditions and a potential therapeutic target.

Adiponectin circulates in the blood at unusually high concentrations (3–30 μg/mL), several orders of magnitude greater than classic hormones and cytokines, corresponding to 0.01–0.05% of total serum protein [3]. Characterized by important variations, plasma adiponectin represents a complex multifactorial trait, influenced by various internal and external genetic and non-genetic factors, as well as physiological and pathological conditions. A large number of linkage and association studies demonstrated the important but not exclusive role of genetic factors in determining the phenotype [4]. Moderate-to-high heritability values, linkage with numerous chromosomal regions, along with genetic variation in the genes *ADIPOQ* and *CDH13* encoding adiponectin and its receptor T-cadherin, which are associated with abnormal concentrations, confirmed the role of genetics as a major determinant of plasma levels and thus adiponectin effects. Genetic factors, however, may have less importance in plasma adiponectin variability compared to other adipokines. Numerous intrinsic and extrinsic factors may modulate circulating adiponectin levels, such as sex, age, cellular differentiation, hormones, fat tissue mass and localization, or diet, physical exercise, and drugs, as well as pathological conditions like obesity or cancer [5].

Dysregulation and altered levels may be associated with various diseases, and adiponectin may represent the missing link between obesity and insulin resistance, and a hallmark of obesity-related pathologies. Though adiponectin is synthesized primarily by the mature adipocyte in the white adipose tissue, obesity is associated with a paradoxical decrease in plasma levels [3]. Moreover, reduced expression of the adiponectin receptors ADIPOR1 and ADIPOR2 determines decreased adiponectin sensitivity, leading to insulin resistance and a vicious cycle [6]. While gene expression is downregulated by various inflammatory mediators in the inflamed adipose tissue, a paradoxical increase in adiponectin levels demonstrating positive correlation with severity and progression may be observed in certain autoimmune diseases with a high inflammatory component [7]. Mendelian randomization analyses could not provide robust evidence for causality in associated diseases and suggest that circulating adiponectin changes could be an epiphenomenon explained by shared etiology [8]. Exosomal packaging of adiponectin with T-cadherin, its role in cellular maintenance and relationship with inflammation, or a potential epigenetic crosstalk may have widespread implications. These mechanisms could also be related to the conflicting observations and the adiponectin paradox of increased circulating levels associated with increased morbidity and mortality [1,9].

Since the discovery of the adiponectin system, studies on gene structure, function and variation have contributed significantly to our understanding of metabolic processes and related diseases, while also suggesting novel intervention strategies [6]. However, data about adiponectin levels are frequently lacking in genetic investigations, and circulating adiponectin assays still present significant limitations. Highly conflicting results of association studies, scarcity of research in certain populations, limited knowledge of rare variants, inadequate study designs, and major gaps in knowledge regarding mechanisms of action, gene–gene or gene–environment interactions make the interpretation of the plethora of data accumulated in the last three decades challenging. Emerging multiomics technologies, functional studies, investigation of large stratified datasets, and controlling for environmental factors could elucidate some important aspects. Understanding the genetic factors influencing plasma adiponectin levels may offer a clearer picture of its role in health and disease.

A state-of-the-art narrative review was conducted on the genetic background of circulating adiponectin levels thirty years after its discovery, at the advent of emerging genomic technologies that may address current knowledge gaps. The literature search was initiated using bibliographical (PubMed, PubMed Central) and genetic (OMIM, UniProt, NCBI, GWAS Catalog) databases, followed by manual reference list screening of pivotal articles, and backward and forward citation tracking. In the case of the bibliographical database search, various terms and their combinations were used (e.g., (“circulating” OR “plasma” OR “serum”) AND (“adiponectin” OR “ADIPOQ”) AND (“heritability” OR “genetic linkage” OR “Genome-Wide Association Study” OR “genetic variation”)), no filters were applied, and all types of materials published since 1995 were included. Publications with no circulating adiponectin assessment were excluded. Sources were selected based on their relevance, historical context and thematic significance. The information was qualitatively reviewed and interpreted with particular attention to contradictory findings for a comprehensive analysis.

## 2. Adiponectin Discovery: A Protein of Many Names

Adiponectin was discovered thirty years ago almost concomitantly by four independent researcher groups using different approaches [2]. In 1995, Scherer et al. identified in differentiating 3T3-L1 adipocytes ACRP30 (adipocyte complement-related protein of 30 kDa), a novel secretory protein with a structure similar to C1q [10]. Constructing the human adipose tissue cDNA library, Maeda et al. sequenced *APM1* (adipose most abundant gene transcript 1), the gene most abundantly expressed in adipocytes that encodes an adipose tissue-specific collagen-like factor with a nectin-like adherent property [11]. Using gelatin-based affinity chromatography, Nakano et al. isolated and characterized GBP28 (gelatin binding protein of 28 kDa) from the human plasma [12]. The symbol *AdipoQ* was designated to the gene by Hu et al. in 1996 [13], while the consensus name adiponectin was proposed by Matsuzawa and colleagues in 1999 [3]. In the next decade, the adiponectin receptors (ADIPORs) ADIPOR1 and ADIPOR2 were identified, including T-cadherin, a crucial binding partner considered the third receptor [1,14,15].

## 3. The Adiponectin System

Adiponectin belongs to the C1q/TNF superfamily of soluble defense collagens, characterized by multimer formation and post-translational modifications resulting in complexes of different molecular weights and distinct functions. In the circulation, the globular fragment is present in minimal amounts, the monomer is undetectable, and 10% is represented by the LMW (low molecular weight) isoform, a trimer of about 90 kDa. The MMW (middle molecular weight) isoform, a hexamer of approximately 180 kDa, and the HMW (high molecular weight) isoform of over 300 kDa, composed of 4–6 trimers, constitute 90% of circulating adiponectin levels [1,2,16].

Once in the circulation, adiponectin must bind to its specific membrane receptors in the target tissues to exert its effects. The adiponectin receptors ADIPOR1 and ADIPOR2 represent the founding members of the PAQR (progesterone adipoQ receptor) family [17]. The two highly conserved ADIPOR paralogs show a homology of 66.7%, while their structural differences explain the different adiponectin binding affinity and thus specific isoform functions [18]. Adiponectin may exert cell type-specific effects dependent on the abundance of its binding partners. ADIPOR1 has high affinity for the globular and low affinity for the full-length form in the muscle, while ADIPOR2 presents intermediate affinity for both forms, and binds preferentially HMW adiponectin in the liver [19,20]. ADIPOR1 and ADIPOR2 lead to the activation of different downstream pathways, mainly, though not exclusively, through AMPK (adenosine monophosphate-activated protein kinase) and PPAR-α (peroxisome proliferator-activated receptor alpha), respectively [1,2,19,20]. The adiponectin receptors, especially ADIPOR2, have intrinsic ceramidase activity [21,22].

The third receptor is T-cadherin, an unusual member of a superfamily of calcium- dependent cell adhesion proteins lacking the cytoplasmic domain. As a glycosyl-phosphatidyl-inositol cell surface-anchored extracellular protein, T-cadherin may act as a binding partner or co-receptor for adiponectin, regulating its bioavailability and tissue accumulation [15]. Genetic association studies strongly suggest its importance as a major regulator of adiponectin levels. T-cadherin binds selectively the HMW and MMW isoforms [15,20]. The accumulation of adiponectin in tissues where the expression of T-cadherin is high and specific leads to the effects dependent on T-cadherin, like cardio-vascular and muscular protection and regeneration [23,24].

## 4. Gene Structure and Function of the Adiponectin System

The adiponectin encoding gene *ADIPOQ* is located on 3q27.3. It spans about 16 kb, containing exons and introns ranging between 0.1 and 4 kb and 1 and 12 kb, respectively (Table 1). The nucleotide sequence shows homology with genes expressed in activated T-cells, encoding the complement factor C1q or collagen type VIII and collagen type X [25,26]. It is expressed predominantly though not exclusively in the adipocytes, primarily but not only in the white adipose tissue, and more in the subcutaneous fat than in the visceral fat [26,27]. Gene expression is induced during adipocyte differentiation, correlates inversely with body fat mass, and visceral obesity is in particular characterized by hypoadiponectinemia [1,2,3].

The *ADIPOQ* regulatory sequences are complex, allowing a sophisticated transcriptional control by diverse endogenous and exogenous signals. The promoter has no TATA box, but contains several crucial CCAAT- and E-boxes, as well as various response elements surrounding exon 1 [28,29,30]. According to deletion analysis, the promoter region between nucleotides −676 and +41 is considered sufficient for basal transcriptional activity [29]. At the end, the gene has a longer 3′-UTR sequence and harbors three *Alu* repeats [31].

The adiponectin receptor genes *ADIPOR1* and *ADIPOR2* were mapped to chromosomes 1 and 12, respectively. While *ADIPOR1* presents ubiquitous expression, highest in the skeletal muscle, *ADIPOR2* is most abundantly expressed in the liver [19,20]. T-cadherin is encoded by the gene *CDH13* located on chromosome 16 in a region considered a major locus linked to plasma adiponectin levels. *CDH13* is expressed in the muscles, cardiovascular and neurological system [27,32].

**Table 1:** Adiponectin and adiponectin receptor genes and proteins [27,31].

Gene Symbol	Name	Genomic Coordinates (GRCh38)	Chromosomal Locus	Number of Exons	Protein Size (Molecular Mass and Amino Acid Number)
*ADIPOQ*	ADIPONECTIN C1q-, AND COLLAGEN DOMAIN-CONTAINING	3:186,842,710–186,858,463	3q27.3	4	26,414 Da, 244 amino acids
*ADIPOR1*	ADIPONECTIN RECEPTOR 1	1:202,940,825–202,958,572	1q32.1	11	42,609 Da, 375 amino acids
*ADIPOR2*	ADIPONECTIN RECEPTOR 2	12:1,691,070–1,788,674	12p13.33	12	43,884 Da, 386 amino acids
*CDH13*	CADHERIN 13 (T-CADHERIN)	16:82,626,969–83,800,640	16q23.3	20	5855 Da, 52 amino acids

*ADIPOQ* expression is influenced by intrinsic and extrinsic factors, such as genetic variation, cellular differentiation, body fat mass and type, age, sex, hormones, the circadian clock, or pathological conditions, as well as diet, physical exercise or certain drugs [5]. Pre-transcriptional regulation through epigenetic mechanisms allows a dynamic control by diverse factors that may have long-term effects. The complex promoter structure containing binding sites for different transcription factors allows the modulation of gene activity by hormones, growth factors or dietary compounds [5,33,34]. The post-translational modifications of the protein are critical for oligomer assembly and multimerization, secretion and stability, and thus adiponectin levels and functions [33,34]. Adiponectin levels are higher in women, during the day, and increase with age [35,36,37]. Gene transcription, protein multimerization and secretion are decreased in various situations like obesity and insulin resistance by pro-inflammatory cytokines, oxidative stress, hypoxia and endoplasmic reticulum (ER) stress. Upregulation is possible by interventions such as diet or exercise, weight reduction or administration of insulin-sensitizers and represents a potential target for novel therapeutic strategies [5,33,34]. Similarly, the expression of *ADIPORs* is influenced by various factors such as age, sex, tissue, body fat or muscular aerobic capacity and can be increased by lifestyle interventions like physical exercise, or may be targeted by experimental therapeutics [38,39].

Circulating adiponectin levels may be influenced by genetic variation directly by *ADIPOQ* variants affecting gene expression, assembly and secretion of functional complexes, or indirectly by variants of the factors controlling these processes [40]. Certain conserved lysine and proline residues in the collagenous domain are critical for post-translational modifications, and the highly conserved cysteine residue located at position 36 (C36) is essential for intermolecular disulfide bonding [1,34,41]. The ER chaperones ERO1-Lα (endoplasmic reticulum oxidoreductase 1 alpha), ERp44 (endoplasmic reticulum resident protein 44) and DsbA-La (disulfide-bond A oxidoreductase-like protein) are crucial for the assembly and secretion of adiponectin [34,42]. If affected, the ER sequestration and degradation of adiponectin may result in hypoadiponectinemia [35,43].

Epigenetic mechanisms allow a dynamic regulation of adiponectin expression [44,45,46,47,48]. Various environmental factors like diet and physical exercise, or the microbiota, as well as pathological conditions like obesity or gestational diabetes, may modulate the epigenetic modifications, influencing gene expression possibly with intra-, inter- and trans-generational impact [44,49,50]. Genetic variants changing DNA methylation sites or miRNA binding sequences may perturb the epigenetic modifications and thus gene expression and circulating adiponectin levels. For example, increased adipocyte DNMT1 expression characteristic for obesity governs the hypermethylation of an *ADIPOQ* promoter region, which may be modulated by genetic variants of the sequence [48]. In fact, the common *ADIPOQ* variants associated with plasma adiponectin rs17300539 or rs266729 are situated in a region undergoing methylation and affect CpG motifs [46].

## 5. Family Studies and Heritability Estimates

Classical family studies suggest an important genetic component in the background of plasma adiponectin levels, although the role of environmental influences could be more important than in the case of other adipokines [51]. The number of heritability studies is limited, and the results are highly variable, related to the important differences in study design and population characteristics. Heritability is often cited as high, although the estimated values vary between approximately 30% and 90%.

Comuzzie et al. undertook the first quantitative genetic analysis for adiponectin levels in 2001. The study was carried out in 1100 adults from 170 families of predominantly Northern European ancestry and detected an additive genetic effect of 46% [52]. In the next study, initiated in non-diabetic Pima Indian siblings, additive genetic influences were found accounting for 39% of the variance of age and sex-adjusted adiponectin [53]. A variance-components analysis on 737 non-diabetic members of 264 nuclear families from Italy found a heritability of only 30% [54]. In a study of 30 monozygotic and 30 dizygotic healthy twin pairs, within-pair, among-components, and intra-class correlation tests consistently demonstrated the increased heritability of adiponectin. The preferred model of a likelihood-based analysis included an additive genetic influence accounting for 88%, independent of BMI [55]. Analysis of the Erasmus Rucphen Family study data from 1258 women and 967 men showed a plasma adiponectin heritability of 55.1% [56]. In 1007 siblings from 360 families of Chinese origin and 352 siblings from 147 families of Japanese origin, a multipoint linkage analysis using a variance components model found a heritability of 70% and 48%, respectively [57].

Interestingly, compared to BMI and leptin levels influenced mostly by genes, the relative contribution of genes appeared comparable to that of shared environment in the case of plasma adiponectin (39% vs. 40%) [51]. Multivariate analysis using data from 228 sibling pairs aged 12–18 years showed higher intra-class correlations for mono-zygotic twins and the BMI-dependent genetic factor explaining only 15% of the adiponectin variation [51].

Heritability of the different isoforms is less investigated. In a family-based sample of 640 non-diabetic White Caucasians from Italy, all forms were found highly heritable (h^2^: 0.60–0.80), but HMW adiponectin showed the highest heritability (approximately 79% vs. 58% in the case of MMW and LMW adiponectin) [58]. Using a variance components approach, the analysis of extended families from Northern Finland with early onset coronary heart disease and low HDL cholesterol revealed a heritability of approximately 53% for total adiponectin, 51% for HMW adiponectin, and 68% for the HMW/total adiponectin ratio [59].

The number of heritability studies in populations other than Caucasian or Asian is also limited. Heritability adjusted for age, gender and BMI was found to be 45% in African Americans coming from 203 families in the Chicago area, and 70% in the Nigerian Yoruba population based on a study of 146 families [60]. In the relatively homogeneous island population of Tobago, investigation of 402 individuals aged 18–103 years belonging to seven multi-generational Afro-Caribbean families demonstrated an adiponectin heritability of 33.2%, while age, sex and BMI explained 23.4% of the variance [61]. The heritability of adiponectin was confirmed only in children in a Saudi population of 403 individuals from 156 families randomly selected from the RIYADH Cohort study [62].

Analysis of the Framingham Heart Study data from 4182 offspring–parent pairs and 1662 sibships demonstrated a quantile-dependent adiponectin heritability increasing with the percentiles of offspring age and sex-adjusted adiponectin distribution, varying between 30% and 79%, depending on the estimation approach [63].

Information about the heritability of sexual dimorphism is scarce and conflicting. In the multi-generational Afro-Caribbean family study, the sex-specific heritability was found significant in men but not in women [61]. In the Framingham Heart Study, heritability was found to be greater in women, in agreement with the characteristic higher concentrations [63]. Heritability computations in the family-based MICROS study showed slightly higher estimates for women than men (65.1% vs. 54.0%). Results confirmed the role of *ADIPOQ* as the only major gene responsible for plasma adiponectin, explaining 6.7% of the phenotypic variance in the population-based KORA Study, or 6.6% of the heritability assessed in the family-based MICROS Study [64]. Genetic variation in the adiponectin gene or other metabolic syndrome loci, however, did not explain the differences seen between men and women [64]. Sexual dimorphism may be related to sex hormones, fat distribution or sex-specific epigenetic programming possibly transmitted in a sex-specific manner [64,65].

Limited information exists about the influence of age and life stages on heritability values, which may decrease over time. A high heritability of 93% (0.93 ± 0.10) for serum adiponectin was found in a cohort of 805 Hispanic non-overweight and overweight children of 4–19 years participating in the VIVA LA FAMILIA Study [66]. Adiponectin levels declined with age, related to sex hormone and growth factor changes, but did not associate with macronutrient intake or daily energy expenditure [66]. Investigation of young and elderly monozygotic and dizygotic twins confirmed the role of a genetic component in the control of both plasma adiponectin and adiponectin receptor expression, modulated by age, sex, abdominal obesity, and aerobic capacity [67].

The significance of family history data was also addressed. In a cross-sectional study of healthy Korean men and women, adiponectin levels were associated with the family history of diabetes independently of obesity and insulin resistance [68].

## 6. Genetic Loci, Genes and Genetic Variants Influencing Plasma Adiponectin Levels

Despite the increased heritability, the genetic background of plasma adiponectin remains incompletely clarified. Though the adiponectin gene is the major determinant, the contribution of *ADIPOQ* variants to the phenotypic variance is apparently reduced [4]. Hypothesis-free research strategies, such as genome-wide linkage scans and genome-wide association studies, suggest that numerous other chromosomal regions, genes and genetic variants may modulate the variation in plasma adiponectin. These investigations often demonstrated the association of the identified genetic factors with diverse physiological traits or pathological conditions related to adiponectin.

The identification of several chromosomal regions linked to the phenotype and the association of circulating adiponectin levels with numerous variants in several genes beyond the adiponectin system suggest a highly complex genetic background. Plasma adiponectin is a quantitative trait determined by a polygenic system, possibly related to tens of regions situated on almost all chromosomes and over 150 genes. In the case of these chromosomal regions, genes and variants, however, the precise mechanisms of action remain largely unknown. Moreover, the contribution of the identified genetic factors to the phenotype may vary across different populations or age groups and may be modulated by environmental factors. The major quantitative trait loci contributing to plasma levels include two regions connected to the adiponectin system: on chromosome 3 harboring the adiponectin gene, and on chromosome 16 in the vicinity of the T-cadherin gene (Table 2).

### 6.1. Linkage Analysis

Evidently, the major region influencing plasma adiponectin variation is located on chromosome 3, where the adiponectin-encoding gene *ADIPOQ* resides, originally identified as an important region and a candidate gene for metabolic and cardiovascular diseases. Genome-wide scans searching for the genetic background of type 2 diabetes and coronary heart disease in a French white and an Indo-Mauritian population recognized its medical importance, identifying 3q27 as a susceptibility locus for diabetes and the metabolic syndrome [71,72]. The first linkage study for adiponectin levels was published in 2001, however, it reported the strongest signals for loci other than 3q27 [52]. The Amish Family Diabetes Study was the first linkage analysis for adiponectin levels that identified the adiponectin locus as the top hit, though with a LOD score of less than 3 [73]. Fine mapping of the 3q27 region identified important sequence variation, and some of the common and rare *ADIPOQ* variants influence plasma adiponectin levels. Most of these variants were also reported as risk alleles for various disorders related to adiponectin.

The first linkage study for adiponectin levels undertaken by Comuzzie et al. in families of Northern European ancestry revealed the strongest signals on chromosome 5 (LOD = 4.06) and on chromosome 14 (LOD = 3.2). The secondary signals, however, included the chromosome 3 region, besides other loci on chromosomes 2, 10 and 17 [52]. The genome-wide linkage analysis of 517 non-diabetic Pima Indian siblings found a maximum LOD on chromosome 9p, which was also present after the inclusion of untreated diabetic siblings totaling 582 cases (LOD = 3 and 3.5, respectively) [53]. The study found further quantitative loci on chromosome 3 (LOD = 1.9), 2 (LOD = 1.7) and 10 (LOD = 1.7) [53]. In an Italian sample, suggestive evidence of linkage was found on 14q13 (LOD = 2.92) after including in the model the adiponectin variant rs1501299 [54]. The study investigated the role of four loci involved in the control of serum levels, including the genes encoding adiponectin and PPARG2, one of the major transcription factors regulating *ADIPOQ* expression [54].

The first linkage analysis describing the *ADIPOQ* locus on chromosome 3q27 as the best hit (LOD = 2.13) was undertaken in 569 non-diabetic participants of the Amish Family Diabetes Study. The study also demonstrated strong association in a dosage-dependent manner with the insertion/deletion polymorphism in the 3′-UTR region, as well as moderate association with rs2241766 and rs1501299 [73].

In the Genetic Epidemiology of Metabolic Syndrome Study, the genome-wide linkage scan in European and Turkish families demonstrated linkage on chromosome 8p23 (LOD = 3.10) in Northern and Western European samples, and also near *ADIPOQ* at 3q28 (LOD = 1.7) in Turkish and Southern European participants [74]. The SNPs most strongly associated with adiponectin were rs3774261 and rs6773957, located within *ADIPOQ* in high linkage disequilibrium (LD). The study also reported association with the *CDH13* variant rs7195409 [74].

A biethnic Chinese and Japanese study reported suggestive linkage on novel loci after adjusting for sex and age. Linkage was found on chromosome 15 (LOD = 3.19) in the population of Chinese origin, and on chromosome 18 (LOD = 2.40) in the population of Japanese origin, besides weaker signals observed on chromosome 3 and chromosome 20 [57]. In the Stanford Asia-Pacific Program for Hypertension and Insulin Resistance Family study of Chinese participants, fine mapping of the QTL mapped on chromosome 15 revealed the strongest association in the case of specific *RYR3* haplotypes [57].

Evidence of linkage with adiponectin was found on chromosomes 2 (LOD = 1.82), 4 (LOD = 2.12) and 11 (LOD = 2.33) in African Americans from the Chicago area, and chromosome 11 (LOD = 2.89) and 17 (LOD = 1.35) in the Nigerian Yoruba population, with a maximum LOD score of 3.21 on chromosome 11 in the combined data [60].

Tejero et al. carried out the first linkage analysis of adiponectin levels in children in 2007 [70]. The investigation involved 466 Hispanic children from 127 extended families participating in the VIVA LA FAMILIA Study with at least one overweight child. The highest LOD score was found on 11q23.2–11q24.2 (LOD = 4.2), followed by 8q12.1–8q21.3 (LOD = 3) and 18q21.3–18q22.3 (LOD = 2.6), though only the prime signal remained unchanged after adjustment for the BMI-Z score. Two other signals suggestive of linkage were reported on chromosome 3 (LOD = 2.1) and 10 (LOD = 2.5), in addition to less important signals on 5q. The results related to 19q and 20q replicated previous findings obtained in adults [70].

The genome-wide linkage scan in the Genetics of Lipid Lowering Drugs and Diet Network Study on 1121 Caucasian individuals from 190 families in Utah and Minnesota, following adiponectin both at baseline and after three weeks of Fenofibrate treatment, demonstrated suggestive peaks (LOD > 2) near *ADIPOQ* on 3q28, as well as on 1p35.2 where the candidate genes *IL22RA1* and *IL28RA* are situated [75]. Further investigation of the same study population found association with *CDH13* and the EGF-receptor signaling pathway [76].

Investigation of genetic and environmental determinants of HMW adiponectin in families with low HDL cholesterol and early-onset coronary heart disease in a Northern Finnish sample found suggestive evidence of linkage on 3q13.2-q24 and 6p21 for the HMW isoform, as well as linkage on 11p15 for total adiponectin levels [59].

### 6.2. Genome-Wide Association Studies

While rare variants may be found more likely by candidate gene strategies combined with deep sequencing of the regions identified by linkage analyses, genome-wide association studies (GWAS) are efficient in identifying common variants in a hypothesis-free approach. Relatively few GWAS have been undertaken for circulating adiponectin levels, and research is constrained by the limitations of phenotype assessment (Table 3). Association study results are often conflicting and incomplete, including replication in different populations. Information remains scarce regarding the biological effects and relationship with the adiponectin system, interactions with genes and other factors, or causality in pathogenic mechanisms. Clearly, the top signals are related to the adiponectin system; however, important population differences exist. While the strongest association with *ADIPOQ* variants was found in populations of Caucasian origin, *CDH13* variation appeared a more important genetic determinant of plasma adiponectin changes in populations of East Asian ancestry.

Genetic variation is significant within the 3q27 region containing the adiponectin gene. Some frequent polymorphisms and rare mutations associate with changes in adiponectinemia, though the information about association with various diseases is much more extensive, and the known variants explain only to a lesser extent variation in circulating protein levels. Through meta-analysis of three genome-wide association studies and validation of lead single nucleotide polymorphisms (SNPs), the GIANT Consortium confirmed that the genetic variants of the *ADIPOQ* region show the strongest association with adiponectin levels [77]. The study identified rs266717 as the lead variant [77]. The study also reported the novel *ARL15* variant rs4311394 associated with lower circulating adiponectin levels and a higher cardio-metabolic risk [77]. Investigating 73 SNPs of various metabolic syndrome loci, Heid et al. found that *ADIPOQ* is the only major gene involved in plasma adiponectin changes, and all the common SNPs investigated in a 50 kb region and covering three LD blocks accounted for 6.7% of the variance [64]. Similarly, analysis combining GWAS of the population-based ERF, KORA and MICROS cohorts, and using samples from 13,795 subjects for the replication stage, yielded only one significant locus corresponding to the adiponectin gene, in both men and women. Nine SNP groups were significantly and independently associated with plasma adiponectin, and the top variant rs17366568 explained 3.8% of phenotype variance [64].

In populations of East Asian ancestry, however, the top signal appeared in the region where *CDH13* is located. GWAS undertaken in a cohort of 26,697 healthy Korean volunteers demonstrated strong evidence for the association of serum adiponectin levels with the *CDH13* region on chromosome 16, besides weaker association with *ADIPOQ* [32]. The top variant found was rs3865188, the major allele increasing gene expression by 2.2 times. In a replication sample, rs3865188 showed even stronger association with the HMW isoform. Investigation of a large cohort of 153,950 participants in the Korean Cancer Prevention Study-II (KCPS-II) by Jee et al. confirmed yet again the significant differences in the genetic architecture of circulating adiponectin measurements in populations of East Asian and European ancestry [78].

The top association with the *CDH13* locus was found in the GWAS carried out in 1776 unrelated Filipino women from the Cebu Longitudinal Health and Nutrition Survey (CLHNS). Similarly, the study detected the strongest signal in the case of rs3865188, besides the weaker *ADIPOQ* signal rs864265, and a novel signal 100 kb upstream, near *KNG1* [79]. The rare *KNG1-ADIPOQ* haplotype (C–T, haplotype frequency: 0.05%) was associated strongly with lower adiponectin levels in both the mothers and the offspring [79]. Conditional analysis for SNPs within the *KNG1–ADIPOQ* gene region showed that the two nearby loci were not completely independent. Though the haplotype spanning the *KNG1–ADIPOQ* locus suggests a more extensive gene region where the potential causal variant/s may reside, investigation of additional variation within a 2 Mb flanking region yielded no results. After adjustment for waist circumference, the associations with adiponectin became stronger for *CDH13* and *ADIPOQ*, but did not change significantly for *KNG1*. *CDH13*, *ADIPOQ* and *KNG1* together explained approximately 7.5 and 8.9% of plasma adiponectin variance in the mothers and their offspring, respectively [79]. Subsequent genotyping detected the uncommon *ADIPOQ* variant R221S largely accounting for the initial signal observed, explaining 17.1% of plasma adiponectin variance [80].

GWAS for HMW adiponectin in the prospective Taiwanese cohort CardioVascular Disease risk FACtors Two-township Study of young onset hypertensive subjects identified *CDH3* as an adiponectin QTL for the first time in Chinese. rs4783244, in strong LD with rs3865188, was found to be associated most significantly with low HMW adiponectin levels and an increased cardio-metabolic risk in men. The polymorphism accounted for 9.28% of the total variance of adiponectin levels, more than the other *CDH13* variants investigated, while association with previously reported *ADIPOQ* and *ADIPOR* variants could not be confirmed [81]. In a study of 530 Taiwanese participants, *CDH13* variants in the promoter and intron 1 such as rs4783244 and rs12051272 were found to influence adiponectin levels independently [82].

rs4783244 was repeatedly confirmed as the top signal in further East Asian samples, explaining 4.1% of total and 6.5% of HMW adiponectin variation, and influencing metabolic health related to the modified adiponectin sensitivity [83]. In Japanese samples, the *CDH13* variants rs3865188, rs4783244 and rs12051272 were found to be associated with higher adiponectin levels, independently of visceral fat, suggesting involvement in an adiponectin resistant status [84,85]. In the Japanese population, the strongest association with adiponectin/HMW adiponectin was found with rs4783244 and in LD with rs12051272 [86].

Since linkage studies suggested that different genetic components might be involved in determining total and HMW adiponectin levels, GWAS was undertaken to clarify the genetic background of the predominant and primary biologically active isoform. In the Nurses’ Health Study (NHS) of individuals with European ancestry, GWAS for serum HMW adiponectin identified association with a novel locus near the non-transmembrane receptor tyrosine kinase encoding *FER* [87]. The association was best represented by rs10447248, a variant situated 170 kb upstream from the gene. The finding was replicated in an independent Italian cohort, the effect accounting for 1.3 and 2% of the variance in the two samples. The study also confirmed that the variants rs822354 and rs3774261 near *ADIPOQ* were related to serum HMW adiponectin levels. rs822354 was reported accounting for 1.1% and 2.5% of the HMW adiponectin variance in the two samples [87].

The European Prospective Investigation into Cancer and Nutrition Potsdam cohort comprising 27,548 middle-aged men and women allowed the identification of an association with rs2943634. The variant located on 2q36.3 in a non-coding region was found associated with a lower risk of coronary artery disease related to slightly higher levels of plasma adiponectin, based on additive models [88]. Pathway analysis trying to decipher the genetic background of plasma adiponectin levels in obese and normal-weight European American women revealed association with *RAC1* (7p22) [89]. RAC1 could be a key element of the involvement of adiponectin in inflammation, cell adhesion and migration, as well as cell survival and apoptosis. The Rac1-centered cell motility pathways were among the top associations tested in the large ADIPOGen Consortium datasets, and replicated in different populations. Additionally, *ADIPOQ* was confirmed among the strongest associations, and the variants rs822387 and rs17300539 reached significance, including in the case of BMI-adjusted adiponectin levels [89]. GPR109A required for the niacin-stimulated adiponectin secretion could also be an important modulator of the phenotype [90]. The role of adiponectin in mediating the effect of major obesity candidate genes is suggested by the Beijing Child and Adolescent Metabolic Syndrome Study. The study found that the *BDNF* variant rs6265 was associated with adiponectin in an additive model, and the rs17782313 polymorphism related to *MC4R* was associated with lower adiponectin levels under a recessive model [91].

To identify novel adiponectin-associated loci, particularly those of importance in East Asians, a meta-analysis of GWAS was carried out in 7827 individuals, followed by two stages of replication in over ten thousand subjects [92]. The study identified genetic variants in 14 loci linked to the variation in adiponectin levels. The results confirmed previously described loci in *ADIPOQ*, *CDH13*, *CMIP*, *GPR109A*, *ZNF664*, and *PEPD*. In addition, novel loci were described on chromosome 10 near *WDR11*-*FGFR2*, explaining 0.6% of the total adiponectin variation, as well as on chromosome 12 near *OR8S1*–*LALBA* [92]. In a Korean sample, the *APOA5* variant rs662799 was found to be associated with lower circulating adiponectin [93].

Combining data across different ethnicities, a large GWAS meta-analysis in 45,891 individuals of mainly European ancestry confirmed the association with previously reported genetic factors such as the *ADIPOQ* rs6810075 and *CHD13* rs12922394 variants, and identified variation in ten novel regions associated with adiponectin levels [94]. Expression analyses on human adipocyte samples revealed that mRNA levels of 18 genes in these regions were associated with adiponectin concentrations. The findings allowed development of a novel multi-SNP genotype risk score to test the association of adiponectin-decreasing risk alleles and metabolic disturbances. Results suggest the involvement of genetic variation in *LYPLAL1* regulating phospholipids on cellular membranes, *GNL3*, *TSC22D2*, *VEGFA* and *TRIB1* involved in vascular biology, *PDE3A*, or *GPR109A* mediating lipolytic effects. Association was also found for the dynein encoding *DNAH10*, *CMIP* involved in T-cell signaling, and *PEPD* catalyzing peptide breakdown [94].

Since common genetic variants were found accounting only for about 5% of plasma adiponectin variance, Spracklen et al. carried out an exome array meta-analysis of 25 studies. The study is the largest available genetic analysis of plasma adiponectin, investigating 265,780 genetic variants in 67,739 individuals of mainly European, but also Hispanic, African American and East Asian ancestry [95]. It identified 30 distinct signals in 20 loci associated with circulating adiponectin levels. Eleven loci were described also in previous studies in or near *LYPLAL1*, *IRS1*, *STAB1*, *ADIPOQ*, *ARL15*, *PDE3A*, *CLIP1*, *DNAH10*, *CMIP*, *CDH13* and *PEPD.* Eight were newly designated loci in or near *KIF9*, *DALRD3*, *FAM13A*, *SLC39A8*, *SNX1*, *GIMAP7*, *RIC8B* and *SLC38A8*. Most of these signals were found to be associated also with at least one obesity or lipid trait. The candidate genes proposed are involved in adipocyte differentiation or bone marrow adipose tissue and include *HDAC9*, *PTH1R*, *AMT*, *NICN1*. The strongest eQTL association signal was found in the case of the cAMP-dependent protein kinase subunit *PRKAR2A*. Gene set enrichment analysis suggested the importance of the ataxin 1 and the glucocorticoid receptor NR3C1 interaction networks [95].

The Metabolic Syndrome in Men (METSIM) study analyzed GWAS data for 9262 men assuming an additive genetic model and testing for association with approximately 16.6 million variants. It confirmed the importance of 12 loci previously identified in European and East Asian populations [96]. The loci *ADIPOQ*, *CDH13*, *IRS1* and *PBRM1* were associated with adiponectin, but only the adiponectin and T-cadherin genes exhibited allele heterogeneity. The four lead signals explaining 7% of circulating adiponectin variation included rs12051272 in the first intron of *CDH13*, rs199938283 near *ADIPOQ*, rs149689033 at 630 kb upstream of *IRS1*, and rs2276824 within a *PBRM1* intron [96].

The large multi-ancestry GWAS meta-analysis of 46,434 individuals from the METSIM cohort and the ADIPOGen and AGEN consortia study for plasma adiponectin levels reported 22 loci, including fifteen loci previously identified [97]. The seven newly described loci were located at or near the genes *CSF1*, *RGS17*, ADRB1, *PDE3B*, *RBMS2*, *HCAR1* and *PHF23*. In the samples of European-ancestry, fourteen additional secondary signals were revealed, located within 1 Mb of the lead variant at the *ADIPOQ*, *CMIP/CDH13*, *HCAR1* and *ZNF664* loci. Among the forty-six putative causal variants identified at seventeen loci, rs2061155, rs1515108, rs1108842, rs998584, rs596359, rs10787516, rs10886863, rs7978610, rs12051272, and rs731839 were the lead variants. Fine-mapping nominated putative causal variants at seven loci, rs16861209, rs998584, rs11045172, rs2925979, rs12051272, and rs731839, representing the lead variants. In general, these variants showed associations with other complex phenotypes, suggesting pleiotropic effects on various physiological traits. Lipid, glycemic, hepatic, hematological and anthropometric phenotypes were primarily affected, and cardio-metabolic diseases and cancer represented the most important pathological consequences. For target gene prioritization at the associated loci, GPScore was derived as a combinatorial likelihood scoring based on measures obtained from eleven gene prioritization strategies and the physical distance to the transcription start site. This allowed identification of the thirty most probable target genes, including the obvious causal genes like *ADIPOQ* or *CDH13*, *IRS1*, *PDE3A*, and *CCDC92*, but also novel genes such as *CSF1*, *RGS17*, *ADRB1*, and *CYP2R1*. Functional association network analysis revealed complex interactions of these genes and underlying pathways connected mainly to adiponectin and insulin signaling through ADIPOR1, ADIPOR2 and INSR, IRS1, and IRS2, respectively. Out of the twenty-two regions, fifteen had one prioritized gene, six had two prioritized genes (i.e., *PBRM1*, *GNL3* at 3p21.1; *ADIPOQ*, *RFC4* at 3q27.3; *NAP1L5*, *FAM13A* at 4q22.1; *FDGFR2*, *WDR11* at 10q26.12; *CTDNEP1*, *ELP5* at chr17p13.1; *PEPD*, *CEBPG* at 17p13.1), and one region had three prioritized genes (i.e., *CCDC92*, *DNAH10OS*, *ZNF664* at 12q24.31) [97]. Lead variants at four loci exhibited evidence of heterogeneity in effect sizes, namely rs17366568 (*ADIPOQ)*, rs12051272 (*CDH13*), rs1515108 (*IRS1*) and rs7978610 (*ZNF664*); these variants were characterized by large differences in allele frequencies and effect in populations of European or East-Asian ancestry [97].

Analysis of large datasets from rapidly growing biobank data allows the investigation of complex multifactorial traits at a new level. Using 405,088 UK Biobank samples, machine learning and a novel highly scalable algorithm offering both state-of-the-art association power and computational efficiency confirmed the primary role of the *ADIPOQ*/*ADIPOQ-AS1* and *CDH13* loci and revealed new candidate genes [98] (Table 3).

**Table 3:** Genetic variants associated with adiponectin levels [99].

Chromosome	Genetic Variant	Risk Allele	*p* < 5 × 10^−8^	Genomic Location (GRCh28)	Mapped Gene	Reference
1	rs2785980	C	+	219,527,177	*LYPLAL1-AS1*, *ZC3H11B*	[95]
	rs2791553	G	−	219,502,700	*LYPLAL1-AS1*	[94]
	rs2061155	C	+	219,491,666	*LYPLAL1-AS1*	[97]
	rs3001032	T	+	219,554,437	*LYPLAL1-AS1*, *ZC3H11B*	[94]
	rs10794657	G	+	24,299,684	*GRHL3-AS1*, *LINC02800*	[94]
	rs13375867	A	−	100,110,377	*SASS6*	[100]
	rs10914455	G	−	31,620,036	*HCRTR1*	[100]
	rs333947	A	+	109,928,142	*CSF1*	[98]
	rs72657272	T	+	30,934,467	*PUM1*	[98]
2	rs2943641	C	+	226,229,029	*MIR5702*, *NYAP2*	[95]
	rs294357	C	+	226,258,723	*MIR5702*, *NYAP2*	[97]
	rs1515110	T	−	226,257,500	*MIR5702*, *NYAP2*	[94]
	rs925735	G	+	226,314,914	-	[94]
	rs6433691	A	−	177,687,230	*PDE11A*, *PDE11A-AS1*	[100]
	rs7560042	A	−	153,599,651	-	[100]
	rs6705747	?	−	68,930,988	*GKN2*, *GKN3P*	[100]
	rs10211067	T	−	98,087,196	*VWA3B*	[100]
3	rs16861209	A	+	186,845,325	*ADIPOQ*	[96,97]
	rs182052	A/G	+	186,842,993	*ADIPOQ*	[94,101]
	rs35469083	T	+	186,856,493	*ADIPOQ*	[102]
	rs141134215	A	+	186,843,412	*ADIPOQ*	[78,101]
	rs199938283	C	+	186,834,681	*ADIPOQ*, *LINC02043*	[96]
	rs4632532	C	+	186,833,893	*ADIPOQ*, *LINC02043*	[96]
	rs73185695	T	+	3:186,834,988	*LINC02043*, *ADIPOQ*	[102]
	rs1648707	C	+	186,833,922	*LINC02043*, *ADIPOQ*	[77]
	rs266717	C	+	186,812,695	*LINC02043*	[77]
	rs864265	G	+	186,836,503	*LINC02043*, *ADIPOQ*	[79]
	rs6810075	T	+	186,830,776	*LINC02043*, *ADIPOQ*	[94]
	rs17300539	A/G	+	186,841,671	*LINC02043*, *ADIPOQ*	[101,103]
	rs17366653	C/T	+	186,853,027	*ADIPOQ*, *ADIPOQ-AS1*	[96,98,101,102]
	rs6773957	A	+	186,855,916	*ADIPOQ-AS1*, *ADIPOQ*	[74]
	rs17366568	G	+	186,852,664	*ADIPOQ*, *ADIPOQ-AS1*	[64,95,104]
	rs17846866	G	+	186,852,957	*ADIPOQ-AS1*, *ADIPOQ*	[96]
	rs62625753	A	+	186,854,237	*ADIPOQ*, *ADIPOQ-AS1*	[98]
	rs562177400	C	+	186,834,680	*ADIPOQ*, *MCF2L2P1*	[98]
	rs10937273	A	+	186,831,906	*ADIPOQ*, *MCF2L2P1*	[92]
	rs143799471	T	+	52,525,026	*NT5DC2*	[98]
	rs66782572	G	+	52,533,601	*NT5DC2*	[98]
	rs2276824	G	+	52,603,470	*PBRM1*	[96]
	rs2590838	G	+	52,588,070	*PBRM1*	[94]
	rs73187787	T	+	186,983,807	*ST6GAL1*	[96]
	rs822354	A	+	186,762,417	*PSMD10P2*, *RNU6-1105P*	[45,87]
	rs11924390	C	+	186,715,310	*KNG1*, *HRG*	[79]
	rs1108842	C	+	52,686,064	*GNL3*	[94,97]
	rs7617025	A	+	150,338,171	*LINC01214*	[97]
	rs13303	C	+	52,523,992	*STAB1*	[95]
	rs758800	T	+	52,495,250	*NISCH*, *STAB1*	[78]
	rs900399	G	+	157,080,943	*LINC00880*, *LEKR1*	[95]
	rs4301033	G	−	150,324,831	*TSC22D2*, *LINC01214*	[94]
	rs266719	T	−	186,783,859	*EIF4A2*	[92]
	rs62294444	G	+	186,777,197	*EIF4A2*, *GPS2P2*	[98]
	rs900400	T	−	157,080,986	*LINC00880*, *LEKR1*	[103]
	rs2276853	A	−	47,240,813	*KIF9*, *KIF9-AS1*	[95]
	rs3087866	C	−	49,017,259	*DALRD3*	[95]
	rs326251	C	−	96,446,567	*MIR8060*	[100]
4	rs13133548	A	+	88,818,977	*FAM13A*	[95]
	rs13131633	C	+	88,818,328	*FAM13A*	[97]
	rs1159918	A	+	99,321,852	*ADH1C*, *ADH1B*	[78]
	rs10517133	C	−	45,362,897	*THAP12P9*	[45,87]
	rs13434995	G	−	55,601,047	*NMU*	[94]
	rs17045969	T	−	165,109,706	*TMEM192*	[100]
	rs17613848	A	−	23,483,389	*RFPL4AP3*	[100]
	rs13111850	C	−	167,343,098	*RN7SL776P*	[100]
	rs10019416	A	−	20,486,088	*SLIT2*	[100]
	rs828154	G	−	17,061,329	*MTND5P4*	[100]
	rs998888	T	−	159,573,678	*LINC02233*	[100]
5	rs4311394	G	+	54,004,832	*ARL15*	[77,95]
	rs6450176	A/G	+	54,002,195	*ARL15*	[94,97]
	rs10447248	A	+	108,580,035	*LINC01023*, *FBXL1*	[87]
	rs13358260	C	−	79,549,888	*Y_RNA*	[87]
6	rs998584	A/C	+	43,790,159	*LINC02537*, *VEGFA*	[94,97]
	rs141574601	G	+	34,199,567	*CYCSP55*, *KRT18P9*	[78]
	rs118106129	G	+	86,798,232	*HTR1E*, *RN7SL643P*	[78]
	rs2523579	C	+	31,360,740	*HLA-B*	[98]
	rs668459	T	−	139,514,552	*LINC01625*, *ATP5PBP6*	[94]
	rs592423	C	−	139,519,556	*LINC01625*, *ATP5PBP6*	[94]
	rs4716055	T	−	9,853,686	*OFCC1*	[94]
	rs6923822	A	−	128,518,325	*PTPRK*	[100]
	rs1283468	A	−	69,328,255	*ADGRB3*	[100]
	rs12211360	A	−	142,798,695	*HIVEP2*	[92]
7	rs10282707	T	+	17,871,415	*SNX13*	[95]
	rs3735080	T	+	150,520,221	*GIMAP7*	[95]
	rs75326924	T	+	80,656,687	*CD36*	[78]
	rs3757791	T	−	127,593,014	*FSCN3*, *GCC1*	[100]
	rs16131	C	−	24,290,218	*NPY*	[100]
8	rs2468677	G	−	139,507,274	*KCNK9*	[87]
	rs2980879	A/T	+	125,469,233	*TRIB1AL*	[94,97]
10	rs10787516	C/T	+	114,054,165	*ADRB1*, *RNU6-709P*	[97]
	rs12771397	T	+	114,022,143	*ADRB*, *NHLRC2*	[78]
	rs10885531	T	−	114,054,633	*ADRB1*, *RNU6-709P*	[94]
	rs3943077	A	+	121,185,572	*LINC01153*	[92]
	rs2429511	C	+	114,041,494	*NHLRC2*, *ADRB1*	[98]
	rs7894696	T	−	78,440,850	*LINC00856*	[100]
11	rs150090666	T	+	14,843,853	*PDE3B*	[98]
	rs61736639	C	−	14,869,595	*PDE3B*	[95]
	rs11023332	C/G	+	14,762,564	*PDE3B*	[94,97]
	rs143019979	A/C	+	14,316,030	*RRAS2*	[98]
	rs2917570	T	−	132,699,866	*OPCML*	[100]
	rs11024603	A	−	18,284,852	*HPS5*	[100]
	rs601727	C	−	100,615,901	*RN7SL222P*	[100]
12	rs7978610	C	+	123,984,025	*RFLNA*, *ZNF664*	[97]
	rs1187415	C	−	124,006,982	*RFLNA*, *ZNF664*	[92]
	rs10861661	C	+	106,780,868	*RIC8B*	[95]
	rs601339	G	+	122,690,196	*KNTC1*, *HCAR2*	[94,97]
	rs491489	T	+	122,693,863	*KNTC1*, *HCAR2*	[98]
	rs7134375	A	+	20,320,824	*LINC02468*, *PDE3A*	[95]
	rs7134375	A	+	20,320,824	*LINC02468*, *PDE3A*	[98]
	rs11057405	A	+	122,297,350	*CLIP1*	[95]
	rs11057353	C	+	123,781,140	*DNAH10*	[95]
	rs7133378	A	+	123,924,955	*DNAH10*, *CCDC92*	[98]
	rs2657888	G/T	+	56,544,599	*RBMS2*	[94,97]
	rs6488898	G	+	123,719,285	*ATP6V0A2*	[94]
	rs7955516	C	+	20,345,102	*PDE3A*	[94]
	rs11045172	A	+	20,317,287	*PDE3A-AS1*, *LINC02468*	[97]
	rs76545872	A	+	20,279,369	*PDE3A-AS1*, *LINC02468*	[78]
	rs144122729	G	+	122,798,508	*CCDC62*	[78]
	rs12369179	T	+	122,479,003	*ZCCHC8*	[98]
	rs10847980	T	−	122,903,375	*VPS37B*, *ABCB9*	[92]
	rs11168618	T	−	48,539,450	*OR5BS1P*, *OR8S1*	[92]
	rs9738548	T	−	128,048,885	*LINC02369*, *LINC02441*	[100]
	rs9989048	T	−	78,911,874	*SYT1*	[100]
	rs10778419	A	−	105,666,324	*CASC18*	[100]
13	rs396318	G	−	56,016,001	*HNF4GP1*	[100]
	rs9315422	T	−	36,481,374	*H2ACP1*, *CCNA1*	[100]
	rs11618218	T	−	51,115,450	*C13orf42*	[100]
	rs1408236	A	−	37,292,760	*RPS12P24*	[103]
14	rs4903705	T	−	78,089,598	*FXNP1*, *NRXN3*	[100]
	rs12887387	T	−	89,729,839	*RN7SKP255*, *CHORDC2P*	[100]
15	rs80039904	T	+	69,901,814	*DRAIC*, *TLRE3*	[78]
	rs1667394	T	−	28,285,036	*HERC2*	[100]
	rs4777845	T	−	93,334,196	*SEPHS1P2*	[94]
16	rs12051272	C/T	+	82,629,683	*CDH13*	[86,94,96,97,98,101]
	rs4782722	T	+	82,638,560	*CDH13*	[96]
	rs3865188	T	+	82,617,112	*CDH13*, *RN7SKP190*	[32,79]
	rs4783244	T	+	82,628,663	*CDH13*	[54,81,92,105]
	rs11646213	T	+	82,609,046	*CDH13*	[95]
	rs7186841	C	+	83,195,109	*CDH13*	[98]
	rs2925979	C/T	+	81,501,185	*CMIP*	[92,94,95,97,98]
	rs9928008	A	+	81,483,993	*CMIP*	[78]
	rs145119400	A	+	84,041,988	*SLC38A8*	[95]
	rs12149200	T	−	628,430	*RAB40C*	[100]
	rs11647294	T	−	77,883,764	*VAT1L*	[100]
	rs17249128	G	−	51,034,374	*LINC02127*	[100]
17	rs17805277	G	+	7,298,434	*EIF5A*, *YBX2*	[97]
	rs5418	A	+	7,281,773	*SLC2A4*	[98]
	rs507506	G	−	7,215,003	*DLG4*	[94]
19	rs4805885	C	+	33,415,217	*PEPD*	[94] [95]
	rs889140	A	+	33,398,094	*PEPD*	[92]
	rs731839	A/G	+	33,408,159	*PEPD*	[94,96,97]
	rs4805881	C	+	33,405,526	*PEPD*	[98]
	rs10415756	A	+	33,403,709	*PEPD*	[78]
21	rs2850066	G	+	35,965,720	*RUNX1*	[100]
22	rs5992838	G	−	17,782,065	*LINC00528*, *MICAL3*	[100]
	rs5752494	C	−	27,127,523	*LINC01638*, *RNU6-1066P*	[100]

## 7. Potential Functional Effects of Genetic Variants of the Adiponectin System

Genetic variation in the adiponectin system identified by GWAS accounts only for about 10% of plasma adiponectin variance, implying missing heritability typical for complex multifactorial traits [96]. Moreover, significant differences exist between the various study results, largely depending on the investigated populations. Conflicting observations are frequently reported, and these may be related to genetic and lifestyle differences and diverse factors such as race, sex, age, body weight, or diseases like type 2 diabetes.

Many genetic variants were identified in the genes encoding adiponectin and its receptors, both in coding and non-coding sequences. The strongest signals for plasma adiponectin are associated with the *ADIPOQ* region on chromosome 3 in populations of European ancestry and the *CDH13* region on chromosome 16 in populations of East Asian ancestry [99]. Genetic variation influencing adiponectin levels is less evident in the case of the adiponectin receptors encoding *ADIPOR1* and *ADIPOR2* [106,107].

There is a large number of association studies with various diseases; however, in these publications, information about the intermediate phenotype is often lacking, while the number of studies following the association with plasma adiponectin levels is more limited. Findings are replicated only for a limited number of polymorphisms, and other factors like age, sex and adipose tissue localization might have more important effects on the variation in the phenotype [56,66]. Rare variants may have a stronger impact (up to 20%) and could explain the missing heritability; however, these mutations are usually population specific [108]. In most cases, the functional consequences and mechanisms of action remain incompletely clarified.

The 3q27 region hosting the *ADIPOQ* gene is of particular importance for plasma adiponectin levels as well as for the cardio-metabolic phenotype. Both common polymorphisms and rare variants were identified in the region, characterized by important genetic variation. In the investigated populations, however, significant differences were described regarding both the allele frequency and effect of specific variants [106,107,108,109,110,111,112,113,114,115,116]. Haplotype analysis of a dense SNP map demonstrated two major haplotype blocks in the *ADIPOQ* gene, one including the promoter polymorphisms associated with increased adiponectin levels, and another within the gene around the junction of exon 2 and intron 2 associated with decreased adiponectin concentration [4,6].

An initial extensive screening of the *ADIPOQ* gene was undertaken by Vasseur et al. in 2002 and detected sixteen SNPs in the investigated French Caucasian sample. The analysis covered a 7.6 kb region, including all exons and introns, 5′- and 3′-UTR sequences, as well as an upstream regulatory region of approximately 1 kb. Both frequent and rare individual variants, as well as characteristic haplotypes were identified, and some of these variants were found to be associated with both altered adiponectin levels and metabolic disease risk [109,110]. A partial screening of the gene in Japanese samples also described frequent and rare variants associated with lower adiponectin levels, but revealed important population differences. While associations described in the French sample did not reach the level of statistical significance in the Japanese population, the associations with common mutations found in the samples of East Asian ancestry were not replicated in the population of European ancestry. Furthermore, most of the identified rare variants were population specific [111,112].

Genetic variation within the coding sequences may cause qualitative changes, altering the amino acid sequence. By perturbing post-translational modifications, multimerization and secretion, certain mutations in specific locations are of particular functional importance and are associated with potential impact on circulating protein levels, isoform formation and effects. Genetic variants located in non-coding sequences may cause quantitative changes by altering the regulation of gene expression. Mutations perturbing transcription factor binding or epigenetic regulation may change the level of gene expression, mRNA and protein quantities. Some of the most important functional variants of the adiponectin system appear due to the genetic variation in promoter sequences. LOC106660625 represents an adiponectin enhancer region, a functional element located on 3q27 in partial overlap with *ADIPOQ*, and contains sequences which may act as enhancers, so its genetic variation may affect adiponectin levels [31]. The role of genetic variants affecting the epigenetic regulation of *ADIPOQ* is less known due to analytical complexity and significant heterogeneity also related to environmental influences. *ADIPOQ-AS1* is an anti-sense RNA gene located on 3q27.3 with three identified lncRNA transcripts possibly involved in the post-transcriptional regulation of adiponectin expression, so the important genetic variation in the sequence could affect protein levels [96].

To evaluate the association of common *ADIPOQ* variants with adiponectin levels and diabetes risk, twenty-two tag SNPs were genotyped across the gene as well as in sequences 20 kb upstream and 10 kb downstream in 2543 participants of the Framingham Offspring Study. In the 5′-sequence, rs822387 and rs17300539 are in strong LD, possibly enhancing transcriptional activity, while in the 3′-region a novel variant rs6773957 was found to be associated with adiponectin levels. The study also confirmed the importance of the coding sequence polymorphism rs17366743, but found no significant association for any other variant [113]. The association study of seventy-seven tagging SNPs of the *ADIPOQ* and *ADIPOR* genes in the Diabetes Prevention Program by Mather et al. described thirteen adiponectin SNPs associated with adiponectin concentrations, reduced by multivariable analysis to rs17366568, rs1648707, rs17373414 and rs1403696 [107]. Investigation of sixty-four tagging SNPs of the adiponectin and adiponectin receptor genes demonstrated no association of *ADIPOR* variants with circulating adiponectin changes. Meta-analysis revealed nine *ADIPOQ* tag SNPs (rs10937273, rs12637534, rs1648707, rs16861209, rs822395, rs17366568, rs3774261, rs6444175, rs17373434) associated consistently with serum adiponectin after adjustment for age, gender and BMI, but the variants explained less than 5% of the serum adiponectin variance [106].

Deep resequencing of *ADIPOQ* in 14,002 samples of European, African American and Indian Asian origin identified 296 SNPs, including thirty amino acid changes and the novel low frequency variant rs17366653 [115]. In the Genetics of Diabetes Audit and Research in Tayside Scotland, seven SNPs were identified via stepwise regression analysis: rs17366653, rs17366743, rs1354091, rs3774261, rs3821799, rs16848727 and rs1868146 had independent effects on adiponectin levels, and together explained 6% of the adiponectin variation [115]. Four non-coding genetic variants within or near the *ADIPOQ* gene (rs17300539, rs17366653, rs3821799, rs56354395) explained 4% of the plasma adiponectin variation by unclarified mechanisms. Investigation of their effect on adiponectin transcription, splicing and mRNA stability in adipose tissue samples demonstrated a 17% increase in expression for rs17300539, a disruption of splicing leading to a seven-fold increase in a non-functional transcript for rs17366653, a 59% decrease in expression for rs56354395, and no effect on gene expression in the case of rs3821799 [117].

Stepwise conditional analysis in the large exome array meta-analysis by Spracklen et al. revealed seven distinct signals in the *ADIPOQ* and two in the *CDH13* region [96]. In the case of the *ADIPOQ* signals, one missense (rs62625753) and one splice mutation (rs17366653) were described in the central region, and further variants were identified in the regulatory sequence. rs76071583 located at about 2.5 kb from the transcription start site demonstrated the strongest effect on transcriptional activity, the A allele associating with a 2.5-fold increased enhancer activity, possibly related to a predicted consensus binding motif for CEBP-α. Functional analysis suggests that the rs13322149-G and rs55958900-A formed haplotype, situated at 135 kb from the *ADIPOQ* promoter and within the first intron of the neighboring *ST6GAL1* gene, may be associated with altered enhancer activity. In the case of the *CDH13* signals, a promoter variant (rs12051272) and a distal intron 1 enhancer variant (rs4782722) may explain the changes in gene expression and thus the influence on circulating adiponectin levels. Considering also the additional signals identified in the *ADIPOQ* and *CDH13* loci, the variants explained 10.3% of the variation in adiponectin levels [96]. rs76786086, in perfect LD with rs115527175, showed the strongest association with subcutaneous adipose tissue expression of *ADIPOQ* and *LINC02043*, the adjacent long non-coding RNA gene. rs17846866 demonstrated the strongest association with subcutaneous adipose tissue expression of *ADIPOQ-AS1*, the anti-sense transcript overlapping portions of the two distal exons of *ADIPOQ* [96].

### 7.1. ADIPOQ Exon and Intron Variants

The central region of the *ADIPOQ* gene was screened extensively, mainly in samples of European and East Asian origin and less often in other populations. The common exon variant does not change the amino acid sequence, and the intron variants usually do not affect the splicing mechanism. Most of the high-impact missense mutations are population-specific rare variants located in exon 3.

#### 7.1.1. Common Polymorphisms

The most common adiponectin mutations studied both separately and as a haplotype are rs2241766 (+45T>G) and rs1501299 (+276G>T) [4,118]. Both variants are situated within the central region of the gene without affecting the protein structure, and in both cases the association studies yielded conflicting results. The +45 exon variant is a silent mutation leading to a synonymous codon (GGT→GGG, G15G), while +276 is an intron variant. It was suggested that most of their effects might be due to LD with the 5′-regulatory sequence variants located at −11391 and −11377. rs2241766 and rs1501299 were investigated in the first association studies in samples of European and East Asian origin with significant differences in minor allele frequencies and effects [6,55,73,109,111,114,116,119,120]. While both polymorphisms were associated with plasma adiponectin changes, insulin resistance and type 2 diabetes risk in the Japanese population, none of them were associated with significant phenotype changes in the investigated French or Italian populations [109,111,114]. Haplotype analysis in healthy Greek women revealed no association of the individual polymorphisms with total or HMW adiponectin levels. It was suggested, however, that those homozygous for the common +45T/+276G haplotype might have lower total adiponectin levels than non-carriers, possibly due to an interaction with body fat [120].

rs2241766 may be a functional polymorphism, although it cannot be excluded that the observations are the result of LD with some other functional variant. Investigated in a Taiwanese population, the steady-state mRNA levels transcribed from the G allele were found higher in the adipose tissue, suggesting that the variant could alter pre-mRNA splicing or mRNA stability [121,122]. Association studies in numerous populations confirmed the initially observed differences regarding the effect and significance [118]. The studies reported effects depending on ethnicity, sex and body fat, as well as apparently different risk alleles. For instance, rs2241766 showed only moderate association with adiponectin levels in the Amish Family Diabetes Study [73], and it was found marginally associated with adiponectin in Filipinos [79]. While the T allele was found in viscerally obese Canadian men, in the healthy Amish population the G allele was found to be associated with decreased adiponectin levels [73,123]. In Finnish adults, a sex-genotype interaction was observed, since significant association was characteristic only for men [124].

The intron variant rs1501299 is situated far from the consensus sequences with no effect on splicing. Still, it might affect the function of neighboring exons, or it could represent only a genetic marker in LD with another functional variant. In the Framingham Offspring sample, rs1501299 was described in strong LD with rs6773957 [113]. It was found to be associated with both increased and decreased plasma adiponectin levels, possibly demonstrating population specific effects, and conferring atherogenic lipid abnormalities in some contexts, while exhibiting protective influence in others [125,126,127]. Hypoadiponectinemia was found to be associated with the G allele in East Asian and certain European populations [54,58,114,119,128], or the T allele in others [118,126,129]. The association was observed in both healthy and obese adults and children [130,131]. Only marginal association was seen in Filipinos investigated in the CLHNS study, and no association could be demonstrated in the African American cohort of the Insulin Resistance Atherosclerosis Family Study (IRASFS) [79,132].

Several other intronic variants were described and investigated in association with adiponectin levels. rs17366568 appears as an important polymorphism associated with adiponectin levels, though only in certain populations of European origin [64,95,97,104,106,107,114,124,133,134]. While rs17366568 explained 3.8% of circulating adiponectin variation in European individuals [64], no association was found in Malaysians [133] or Black women from southeastern USA [134]. No transcription factor binding sites were identified in the sequence and no effect on splicing could be detected. Though the regulatory potential in the region seems low, the findings could still be related to a functional element located in the immediate vicinity of the variant. In an adipose tissue-specific analysis, three SNPs located immediately up- and downstream of rs17366568 were predicted to affect 10, 6 or 4 transcription factor binding sites [64]. In the Finnish Diabetes Prevention Study, rs17366568, rs16861210 and rs6773957 were found strongly associated with baseline serum adiponectin levels in a dose-dependent manner; in the case of rs17366568 and rs6773957 LD was suspected. rs17366568 and rs16861210 are both located in the same intron but no strong LD was observed, suggesting independent signals [124]. In Asian Indians, the association between rs17846866, a genetic variant located in the first intron at +10211, and hypoadiponectinemia as well as obesity and type 2 diabetes risk was identified [135].

#### 7.1.2. High-Impact Rare Variants

Variation in the polypeptide chain in key positions may have significant effects on adiponectin structure, secretion and clearance. Since these variants may alter plasma levels and isoform functions, they are associated with potential pathological implications. The selection of individuals with the lowest adiponectin levels for sequencing may lead to a significant enrichment of non-synonymous changes. The identification of such mutations is of particular theoretical and practical importance. Natural genetic variants described in patients with extensive phenotyping associated with in silico modeling and in vitro functional studies may offer important insight into adiponectin biology. Detection of these variants in clinical practice may contribute to more personalized management.

The non-synonymous coding sequence mutations leading to amino acid changes are usually rare, population specific, and reported as heterozygous genotypes. The findings related to their association with low adiponectin levels and cardio-metabolic disease risk are inconsistent. In the initial studies, G84R, G90S, R92X and Y111H were reported in the French population, while G84R, R112C, I164T, R221S and H241P were found in the Japanese population. Of these missense variants, G84R, G90S, Y111H, R112C and I164T were found to be associated with hypoadiponectinemia [6,109,112].

Missense variants causing changes in the amino acid sequence may lead to alterations of the spatial organization, isoform formation, secretion or interaction with the receptors. Mutations associated with phenotype changes usually alter the collagenous domain (e.g., G84R, G90S), the hinge region between the collagenous and globular domains (e.g., Y111H, R112C), or the globular domain (e.g., I164T). The highly conserved lysine residues in positions 68, 71, 80 and 104, as well as the proline in position 94, are crucial for isoform formation, so their changes may have significant functional consequences [136,137,138].

In silico analysis using SIFT, PolyPhen-2, PROVEAN, Mupro and I-Mutant 2.0 predicted 14 deleterious and 38 less stable mutations in the case of the fifty-eight non-synonymous point mutations (55 missense and 3 nonsense) identified in SNP databases [139]. About one third of these mutations are located in the collagenous domain necessary for multimerization, and about two-thirds of these mutations affect the C-terminal globular domain responsible for higher order complexes and interaction with the receptors. Ten variants appear both deleterious and reducing protein stability, namely c.133G>C (G45R), c.143G>A (G48D), c.163C>T (R55C), c.223G>T (G75C), c.250G>A (G84R), c.268G>A (G90S), c.334C>T (R112C), c.335G>T (R112P), c.595G>A (G199S) and 626A>G (D209G). Nonsense mutations causing truncated protein structures may abolish the secretion of functional adiponectin. Of the three nonsense mutations, c.274C>T affects the collagen region, while c.635G>A and c.658G>T change the complement component C1q domain [139].

The N-terminal end contains the signal sequence corresponding to the first 18 amino acids and the variable region [12]. Exon sequencing of 30 adolescents chosen from a school-based cohort based on serum adiponectin levels led to the identification of the methionine initiation skip (MIS, c.130G>A) and P32L (c.95C>T) variants associated with very low (<1%) adiponectin levels [140]. The MIS mutation was predicted to eliminate the first 39 amino acids and the signaling peptide. The mutation carrier had the lowest adiponectin concentration with little HMW and no VHMW (very HMW) isoform, but exhibited normal adiposity and insulin, so the variant was considered benign [140]. P32L is within the hypervariable region, located in a site probably not highly conserved. Similar to MIS, P32L caused very low adiponectin levels characterized by lower HMW and very low VHMW adiponectin, but the mutation carrier was overweight and hyperinsulinemic, so the variant is considered possibly damaging [140]. The reduced functionality may be due to the proximity of a conserved lysine (K33) and C36 critical for disulfide bonds.

As a terminal post-translational modification within the variable domain, sialylation takes place after multimerization and before exocytosis. Sialic acid content may affect circulating adiponectin stability and thus half-life. Site-targeted mutagenesis revealed the primary importance of certain threonine residues for sialylated isoform formation, especially in the case of T22 and to a lesser extent in the case of T21. If these residues are abolished, it results in accelerated plasma clearance [141].

For the formation of multimers larger than a trimer, a disulfide bond is required through an amino-terminal cysteine. The C36S mutation leads to the secretion of adiponectin only as a trimer and abrogates the effect on the AMP-activated protein kinase pathway in hepatocytes. This demonstrates that the C36 residue is required for MMW and HMW isoform formation and ER retention through thiol-mediated covalent attachment to its chaperones [136].

Screening for low adiponectin levels and sequencing the adiponectin gene in a cohort of young subjects led to the identification of M40K (c.119T>A) in a heterozygous male with significant cardio-metabolic dysfunction [142]. Conserved between species, M40 is located near the end of the variable region, in the vicinity of C36. The M40K mutation may disrupt quaternary folding, and it impairs secretion, which explains the significant reduction in HMW adiponectin and mainly trimeric adiponectin. All mutation carriers (the proband and three relatives) presented a marked reduction in serum HMW/total adiponectin ratio and features of the metabolic syndrome. In the adipose tissue, adiponectin mRNA and protein levels were increased, and the expression of the adiponectin receptors was downregulated, especially in the case of *ADIPOR1*. The proband was small for gestational age at birth, possibly related to the reduced maternal HMW levels associated with the carrier status of the mother. Obesity developed during adolescence, possibly due to the depletion of HMW adiponectin associated with increasing testosterone levels. The early-onset metabolic phenotype also included insulin resistance, dyslipidemia, liver steatosis, hypertension, increased carotid intima media thickness, and type 2 diabetes manifested in young adulthood [142].

The conserved collagen-like region between amino acids 42 and 107 contains key residues crucial for post-translational modifications, which determine the efficiency of multimer formation and secretion. Since site-specific mutagenesis impairs HMW multimer formation, adiponectin multimerization is considered dependent on the conserved lysine residues K65, K68, K77 and K101 of the collagenous domain [136,137].

The IRASFS investigations were carried out in populations disproportionately affected by type 2 diabetes and were characterized by high-quality metabolic phenotype assessment. Two high-impact rare (≈1%) ethnic-specific *ADIPOQ* missense mutations were described: G45R in Hispanic Americans and R55C in African Americans [108,132,143,144]. The lack of consistent association between the evaluated metabolic traits and these variants that significantly decrease adiponectin levels emphasized the need of reassessing the role of adiponectin changes in metabolic pathways, whether a cause or a consequence [132]. The findings also suggested the importance of further isoform investigations.

The missense mutations G45R and R55C located in the collagen-like domain cause the reduction in adiponectin levels to approximately 15–20%, and yet the carriers develop no significant metabolic dysfunction. The absence of HMW and a marked reduction in MMW adiponectin were associated with a concomitant approximately two-fold increase in the LMW isoform. It was suggested that LMW adiponectin may compensate for some of the lost or reduced higher-order multimer function, contradicting the presumed biological importance of the HMW isoform [144]. A possible functional compensation for the lack of HMW adiponectin, however, is not observed in carriers of other variants characterized by metabolic perturbations despite normal LMW and MMW levels [144].

The rare G45R (c.133G>C, rs200573126) mutation is located 523 bp from the intronic variant rs17366568, and interferes with the assembly and secretion of HMW adiponectin. It explains a strong linkage signal on 3q27 with an LOD score of 8.21, accounting for 8.8% of the plasma adiponectin variance in Hispanic Americans [108]. The surprising absence of association between significantly reduced adiponectin levels and changes of the insulin sensitivity index could suggest that adiponectin is not a direct effector of insulin resistance, or a physiological compensation for the low levels of adiponectin may exist in the mutation carriers [108].

The rare R55C variant (c.163C>T, rs143606172) was found only in African Americans, and not in Caucasians or Hispanic Americans [108,132]. It disrupts the collagenous domain characterized by highly conserved Gly-X-Y motifs and leads to the inability of HMW adiponectin formation. Carriers manifest very low adiponectin levels, but no evidence of significant changes in the glucose homeostasis and adiposity phenotypes studied, putting into question the role of adiponectin in type 2 diabetes development [108,132]. Introducing a new cysteine nineteen amino acids downstream from C36, R55C might affect disulfide bond formation [140]. Carrier adolescents were found to be insulin resistant and obese, and they presented low levels of adiponectin with little HMW and no VHMW, but a significant amount of LMW adiponectin [140]. At the same position, the substitution R55H described in Thai type 2 diabetes patients was found to cause impaired protein multimerization and significantly reduce HMW adiponectin levels, but not disturb trimeric and hexameric adiponectin formation [139,145].

The amino acids G84 and G90 are both located in Gly-X-Y repeats within a highly conserved region of the collagenous domain, important for triple helix formation and higher order multimerization [136,145]. In the case of the mutations G84R (c.250G>A, rs199646033) and G90S (c.268G>A, rs62625753), substitution of the smallest amino acid glycine with serine or the electrically charged arginine may disrupt the collagen triple helix [6,136]. The changes may affect HMW multimer formation without disturbing the formation of the trimeric and hexameric forms [6,135]. Though situated far from the disulfide bond forming C36, the multimerization defect could still be related to conformational changes by concealing the free thiol and preventing the interaction with other hexamers or disturbing the interaction with ER chaperones [5,145].

The G84R and G90S variants are rare and characterized by population differences. Initially, G90S was described in the French population, but it was not found in the Japanese population, while G84R was reported in both populations [109,111]. The phenotype is characterized by hypoadiponectinemia with altered HMW but normal trimer and MMW formation, associated with metabolic effects, including altered adiponectin functions in the liver, and increased diabetes risk [96,109,111]. Interestingly, G90S was found to be associated with abnormal aggregation of tau proteins and contributing to cognitive degeneration [146].

The R92X nonsense mutation causes the lack of the globular domain and no detectable monomers, possibly due to an unstable truncated mRNA that prevents translation, or translation of a rapidly degraded polypeptide [5,109,135]. The mutation reported in one heterozygous patient with no documented hypoadiponectinemia, insulin resistance or diabetes might suggest autosomal recessive inheritance, if such a monogenic disease exists [109].

A unique 10 bp deletion (CCCGAGGCTTT→C, rs777532127) was identified at G93, leading to a frameshift mutation and premature termination of translation after 73 amino acids. The mutation was reported in a family with multiple members affected by type 2 diabetes and end-stage renal disease, low adiponectin levels (<20%), the lack of HMW isoform, and increased ceramide levels [147]. Functional studies demonstrated an interaction between the wild-type protein and the truncated protein resulting in decreased stability of the normal protein, which suggests a dominant negative effect possibly explaining the severe adiponectin deficiency [147].

The variants changing the amino acids in positions 111 and 112 affect the hinge region between the collagen and globular domains. The mutations may perturb the assembly of collagen-like homo-trimers in bundles and change the spatial organization of the protein. The structural changes might interfere with post-translational modifications such as glycation of lysine residues essential for insulin sensitivity modulation, alter excretion, or modify interactions [6]. While variation in the less conserved 111 site is considered benign, mutation at the highly conserved 112 position is considered pathogenic and may cause significant multimerization and secretion defect [6,109,136]. These variants also appear to be population specific. Initially, Y111H was identified in the French population, but it was not found in the Japanese samples, while R112C was described in the Japanese population, but it was not found in the French samples [109,112].

The observations are inconsistent regarding the association of Y111H (c.331>C, rs17366743) with reduced adiponectin levels. The mutation was first described in the French population, associated with hypoadiponectinemia and increased type 2 diabetes risk [109]. The relatively conservative tyrosine-histidine substitution at the less conserved position 111 does not impair multimer formation, though it could cause subtle structural changes and secretion defects explaining the phenotype [109,136,140,145].

In contrast, the less conservative arginine–cysteine substitution at the highly conserved position 112 severely affects multimer formation and secretion [109,136,140,145]. Screening for ADIPOQ mutations in the Japanese population led to the identification of the pathogenic missense mutation R112C (c.334C>T, rs121917815) in a non-obese patient with significant hypoadiponectinemia (<30%), coronary artery disease, lung thrombosis and autoimmune disease [26]. Out of four children, a daughter carried the mutation, and also presented low adiponectin levels [26]. R112 is located in the globular domain, on the contact surface near the basal side. Functional analysis in NIH3T3 fibroblasts demonstrated that the R112C mutation prevents stable LMW trimer assembly, resulting in impaired secretion and thus adiponectin deficiency [26,136]. The variant causes no oligomeric class formation, likely due to the breaking of H-bonds between monomers. While the R112C variant protein was found aggregated in cell lysate and undetectable in culturing media, all three oligomeric forms were present in the case of the R112H variant. R112H affects the same position but by a more conservative mutation, replacing two positively charged amino acids [145]. Hetero-oligomers between these mutants and the wild-type protein may be formed, causing a secretion defect of normal adiponectin by sequestration in the co-aggregated forms in the context of a possible dominant negative effect [145].

R131 represents another conserved position. The R131H (c.393G>A) variant was identified only in diabetic patients but not in non-diabetic controls [145]. The mutation is likely implicated in the pathogenesis of type 2 diabetes by impaired adiponectin oligomerization and secretion. In silico modeling revealed the alteration of H-bond-forming patterns within the globular domain that may perturb the formation of adiponectin trimers. This variant may also have a dominant negative effect, decreasing the secretion of the normal protein by forming aggregated complexes with the wild-type protein [145].

Situated on the contact surface of the globular domain, Y159 has an important role in the formation of the hydrogen bond network between trimers [145]. Genetic variation such as Y159D (c.475T>G) may cause susceptibility to degradation within the cell, impairing secretion and causing hypoadiponectinemia with barely detectable multimer levels [136].

I164 represents a highly conserved hydrophobic residue on the contact surface of the globular domain. The I164T variant (c.491T>C, rs185847354) is considered pathogenic, causing significant hypoadiponectinemia (≈30%) via impaired LMW trimer assembly and secretion [136]. The mutation was found to be associated with diverse cardio-metabolic phenotypes, including type 2 diabetes, hypertension and atherosclerosis [6,112,148,149]. The hydroxyl group of threonine, instead of the hydrophobic side chain of isoleucine, may alter the hydrophobic core in adiponectin monomers and disturb trimerization, so no oligomeric classes form, and secretion defect results [145].

Variation in the C-terminal part appears less important. Initially, it was thought that these variants do not affect a critical region in the protein structure, and they have no influence on adiponectin levels [6]. The mutations R221S (c.661C>A, rs138773406) and H241P (c.722A>C, rs141205818) were identified in the Japanese population, but they were not found in populations of European ancestry. The variants did not associate with changes in circulating adiponectin levels or clinically obvious phenotype alterations. It was suggested that the mutations affect a less conserved region in the globular domain but not the contact surface, so they do not perturb oligomerization, multimer formation and secretion [6,111,112,135,145]. A subsequent genotyping of the CLHNS sample, however, found that the uncommon R221S variant explained 17.1% of plasma adiponectin variance [80].

### 7.2. Potential Regulatory Sequence Variants of ADIPOQ

#### 7.2.1. Genetic Variation in the Promoter Region

Genetic variation in the 5′-regulatory region may change transcription factor binding and epigenetic regulation, crucial for mediating the influence of various signals from the internal and external environment or physiological and pathological conditions. Haplotype analyses of missense mutations within the coding region and variants within the 5′-regulatory sequence suggest a synergistic effect on the phenotype [6].

In the promoter region, there are two haplotype blocks defined by rs2241766 and rs1501299, or rs266729, rs16861194 and rs17300539. The allele frequencies and effects show significant racial differences. *ADIPOQ* promoter variants appear important primarily in populations of Caucasian and African origin, while their significance could not be confirmed in other populations. In the CLHNS study of Filipino mothers, for instance, rs17300539 was found to be monomorphic, rs16861194 showed no association, and rs266729 demonstrated only modest association with plasma adiponectin [79].

Within the 5′-regulatory non-coding sequence, the polymorphisms rs17300539 (−11391G>A) and rs266729 (−11377C>G) were first described in a French sample, identified as a GG risk haplotype associated with lower circulating adiponectin and type 2 diabetes risk [109]. This association, however, was not confirmed in a Japanese sample, probably related to the low allele frequency [6,111]. The promoter variants rs266729, rs17300539 and rs16861194 (−11426C>G) are located within an 80 bp sequence of the enhancer region. Haplotype analysis demonstrated association with circulating adiponectin levels, and the loss of basal and rosiglitazone-induced promoter activity in 3T3-L1 adipocytes in the case of the three minor alleles [150]. However, a population-based epidemiological investigation of the MONICA/KORA S123 and KORA S4 cohorts demonstrated a lack of this haplotype [150]. rs16861194 was in particular associated with reduced promoter activity and hypoadiponectinemia [150].

One of the *ADIPOQ* variants showing the strongest association with adiponectin levels is rs17300539 (−11391G>A). The A allele was repeatedly found to be associated with higher adiponectin levels in various populations of Caucasian origin, both adults and children, with or without obesity [56,58,65,109,110,113,131,132,151,152]. In the IRASFS African American cohort, the low-frequency rs17300539 was one of the most important variants associated with plasma adiponectin changes, similarly to populations of European ancestry [132]. In populations of East Asian ancestry, rs17300539 demonstrated no significance: it was found to be extremely rare or absent, and it was not associated with functional consequences [79,111,149]. Candidate gene analysis in a Spanish Mediterranean population of the four most frequently investigated *ADIPOQ* variants, namely rs17300539, rs2241766, rs1501299 and rs17366568, showed a strong and additive association only in the case of rs17300539 [100].

Functional consequences were found inconsistently, and the effects might be related to the transcriptional or epigenetic regulation. Presence of the rs17300539 minor A allele increased promoter activity in COS-7 cells, which, however, do not represent an adipocyte cell line [131]. In adipose tissue samples, a 17% increase in gene expression was associated with the rs17300539 minor allele [117]. rs17300539 together with the other non-coding variants rs17366653, rs3821799 and rs56354395 explain 4% of plasma adiponectin variation by affecting *ADIPOQ* transcription, splicing and stability. rs17300539 might increase the binding affinity of transcription factors or interrupt a potential regulatory element. CHiP analysis revealed that rs17300539 lies in a highly conserved region containing binding sites for the transcription factors ZNF217, EP300, GATA3 and FOXA1. The effects observed, however, may be driven by other variants located in the nearby 5′ (rs73185695, rs57056768, rs822387), intergenic (rs73185691) or intronic (rs73185701, rs73185702, rs16861209 and rs16861210) sequences [117]. Haplotype analyses repeatedly suggested the existence of functional interaction between rs17300539 and rs16861194. The rs16861194 minor allele was found significantly reducing DNA binding and basal promoter activity, though the association with adiponectin levels was not always confirmed [81]. The binding factors remain largely unclear, though rs17300539 and rs16861194 could interfere with putative homeobox transcription factor binding, NKXH and CART, respectively [150]. A higher inducibility by Rosiglitazone was seen in the case of the rs17300539 minor allele only in combination with the major allele of rs16861194, which may have pharmacogenetic importance [150]. Moreover, rs17300539 is located in a distal promoter region characterized by DNMT1-dependent hypermethylation related to obesity, and alters a CpG dinucleotide that may influence DNA methylation, and thus transcription [46]. Significant association of rs17300539 with CpG island methylation was also observed on the fetal side of the placenta, sensitive to maternal glucose levels [44].

Studies of rs266729 (−11377C>G) in various populations suggest ethnic-specific importance and effects. Initially, low adiponectin levels and metabolic disorders were reported in association with the G allele, but later also with the C allele in other populations [4,73,109,118,150,151,153]. A Mendelian randomization study in a multiethnic population assembled in Canada from the SHARE/SHARE-AP cohorts concluded that the minor G allele was associated with lower adiponectin levels [154]. In Korean adults, the G allele was found to increase the risk of obesity and low adiponectin levels without associating with significant changes in BMI or adiponectin alone [155]. In the CLHNS study of Filipino mothers, only modest association with plasma adiponectin could be demonstrated [79], and no association was found in the IRASFS African American cohort [132]. A meta-analysis confirmed that G allele carriers had lower levels of adiponectin and high-density lipoprotein cholesterol than CC homozygotes [156]. In the Finnish Diabetes Prevention Study, however, the C allele was associated with an increased adjusted hazard ratio of developing type 2 diabetes, without significant association with adiponectin levels [124]. It was also suggested that a BMI–genotype interaction could characterize the association between rs266729 and adiponectin levels or diabetes incidence [113]. While some functional studies suggest no influence on transcription [131], according to haplotype analysis and bioinformatics prediction, the rs266729 variant could affect *ADIPOQ* transcription, possibly modifying a zinc finger transcription factor-binding site [150,157]. The variant might alter the sequence of one of four SP1-binding sites in the promoter region. Both rs17300539 and rs266729 reside in a distal promoter region located about 1 kb upstream of the transcription start site, characterized by DNMT1-dependent hypermethylation related to overnutrition and inflammation in obesity. Since each polymorphism alters a CpG dinucleotide, both variants could impact DNA methylation and thus *ADIPOQ* expression related to lifestyle changes and disease states through epigenetic mechanisms [46].

#### 7.2.2. 3′-UTR Variants

The 3′-UTR sequence may act as a molecular hotspot for pathology primarily through miRNA and RBP binding [158]. In the case of *ADIPOQ*, several polymorphisms were identified in the region, and some of these variants were found to be associated with circulating protein levels with varying importance across populations. Functional consequences may be related to post-transcriptional gene expression regulation through changes in mRNA stability and translational efficiency. However, certain observations suggest that the effect may not be independent but related to other functional variants [100].

rs6773957 was described as one of the polymorphisms most strongly linked to adiponectin levels, mainly in populations of European origin, the minor allele associating with lower circulating adiponectin and metabolic disturbances [58,74,84,113,124]. In the Framingham Offspring Study that extended mapping up to 10 kb downstream after the gene, rs6773957 represented the strongest signal associated with higher adiponectin levels [113]. However, rs6773957 was found in moderate LD with the nearby rs6444175 polymorphism located further downstream, and in strong LD with rs1501299. It could not be determined which polymorphism was responsible for the association, though regression analysis indicated that the effect of rs6773957 was not independent and could be related to the functional promoter variation [113]. In the Finnish Diabetes Prevention Study participant adults with IGT and overweight, rs6773957 was strongly associated with adiponectin levels and body weight in a dose- dependent manner at baseline and after a 4-year follow-up [124]. In multiple SNP analyses the significance of rs6773957 was lost, indicating LD with another variant, most likely rs17366568 [124]. The Beijing Child and Adolescent Metabolic Syndrome study confirmed the association of rs6773957 with lower adiponectin levels and a metabolically unhealthy phenotype in normal weight and obese children aged 6–18 years [159]. The study also observed a novel gene-diet interaction, since the G allele was associated with greater metabolic risk in case of unfavorable dietary patterns, but not in individuals with healthy patterns [159]. The variant rs6444175 located further downstream near the 3′-UTR was found in intermediate LD also with rs3774261. rs6444175 might explain 3.5% of the total variance of plasma adiponectin levels, and could be important for insulin sensitivity, and the development of obesity and type 2 diabetes [106,160].

The +2019 mononucleotide (A) insertion/deletion polymorphism (rs56354395) demonstrated high allele frequency (30%) and strong association with adiponectin levels in a dosage-dependent manner in the Amish Family Diabetes Study. Deletion heterozygosity increased adiponectin levels by 1.3 ± 0.5 μg/mL, while deletion homozygosity increased adiponectin levels by 3.0 ± 0.8 μg/mL [73]. Mature transcript analysis in the adipose tissue demonstrated a reduction in expression in carriers of the insertion allele. The effect might be attributed to a more open conformation rather than to altered miRNA action (binding sites predicted for miR-581, miR-106b*) [117]. Interestingly, in homozygous form (del/del) the variant associated with increased adiponectin levels was found more prevalent in long-lived males in a relatively homogeneous cohort of Ashkenazi Jews, possibly related to an effect on mRNA stability or translational efficiency [161]. rs56354395, however, was found in almost complete LD with the common +45T/+276G haplotype [114,132].

### 7.3. Circulating Adiponectin Levels and the Genetic Variation in Adiponectin Receptors

Besides *ADIPOQ*, the *CDH13* locus is the most important region associated with adiponectin levels, especially in populations of East Asian ancestry, whereas genetic variation in the *ADIPORs* appears less important.

Relatively few studies investigated *ADIPOR1* and *ADIPOR2* variants in relation to adiponectin levels, and usually no association was found [57,74,106,107]. Inconsistently, however, certain variants were found modulating adiponectin levels, like rs10920531 (A>C, in the 3′-flanking region) in the case of *ADIPOR1*, and rs16928751 (+795G>A), rs767870 (+870C>A), rs11061971 (+963C>T) in perfect LD in the case of *ADIPOR2* [162,163].

Variants of the third receptor T-cadherin may have an important effect on circulating adiponectin levels, especially in populations of East Asian ancestry. Several common and rare variants were described in association with altered adiponectin levels, including rs4783244, rs38655188, rs8060301, rs12596316, rs8047711, rs7193788, rs12051272, rs12443878, rs62040565, rs12444338, rs12922394, rs7195409, rs2239857, rs77068073, and rs113460564. These variants are located primarily in a haplotype block covering the 5′-regulatory region and the first intron, and some of them are related to increased promoter activity [32,57,74,79,81,82,83,84,85,86]. The paradoxical association of higher adiponectin levels with worse metabolic profile may suggest compensatory altered blood levels in response to adiponectin resistance. *CDH13* variants are considered a key driver of the HWM/total adiponectin ratio in populations of East Asian ancestry, and some of these variants were found to be associated with primarily cardio-metabolic pathology [81,83,164,165,166].

The first GWAS of adiponectin levels undertaken in East Asian populations discovered rs38655188 (A>T) in *CDH13* as the top signal in Korean volunteers. The variant was characterized by an even stronger association with the HMW isoform, in complete LD with the promoter variant rs12444338, showing a 2.2-fold increase in expression in vitro [32]. Similarly, in Filipino women from the CLHNS analysis, the strongest signal was rs3865188, in limited LD with rs7195409 [79]. Genotyped in two French samples from the DESIR and DIABHYCAR studies, rs3865188 and rs11646213 demonstrated different genotype distributions [166]. The rs3865188 variant was found to be associated with higher adiponectin levels and lower BMI, fatty liver index and type 2 diabetes risk, while the rs11646213 variant was found to be associated with lower adiponectin levels and higher BMI and type 2 diabetes risk [166].

Analysis of combined data from Singaporean Chinese, Korean and Japanese cohorts revealed the significance of rs4783244 (G>T) [83]. rs4783244 represented the top signal associated with total adiponectin levels, and demonstrated an even stronger association with HMW adiponectin levels and the HMW/total adiponectin ratio. The variant explained 4.1–5.5% of total adiponectin and 6.5–8.3% of HMW adiponectin variation in the different cohorts [83]. Since metabolic health was better than expected based on circulating adiponectin levels, an increased binding of HMW adiponectin to the CDH13 receptor was suspected in the case of the T allele, explaining the increased tissue sequestration and reduced blood levels. *ADIPOR* upregulation caused by chronic hypoadiponectinemia might counterbalance low adiponectin levels and explain the lack of metabolic disturbances in unadjusted analyses [83]. The rs4783244 genotype was found to be associated with the prognostic significance of HMW adiponectin levels: all-cause mortality increased linearly with the number of G alleles [165].

rs4783244 is located in the first intron, in a 30 kb haplotype block covering the promoter and first intron region, in moderate LD with rs12051272. rs12051272 was also found to be associated with adiponectin and in particular HMW isoform levels in the Japanese population [86]. In Han Chinese samples from Taiwan, the rs12051272 variant and ATTT haplotype of the rs11646213 major allele and rs12444338, rs4783244, and rs12051272 minor alleles were most significantly associated with lower circulating adiponectin levels [82]. Investigation of the rs4783244 and rs12051272 variants by luciferase reporter assay demonstrated increased enhancer activity in the case of the GG haplotype and decreased enhancer activity in the case of the TT haplotype [105]. In young onset hypertensive subjects from Taiwan, three variants responsible for hypoadiponectinemia showed genome-wide significance, namely rs4783244, the intronic variant rs8047711, and the promoter variant rs7193788 [57]. The *CDH13* variants rs3865188, rs4783244, and rs12051272 were found to be associated with adiponectin levels independently of visceral fat assessed by computed tomography in a Japanese sample, confirming their role in an adiponectin-resistant status [84,85].

Certain *CDH13* variants might influence the level of methylation at neighboring CpG sites, causing altered gene expression by modulating epigenetic regulation. Investigation of the HYPertension in ESTonia/Coronary Artery Disease in Czech samples led to the identification of the rare variant rs113460564 (A>C) as a highly significant meQTL for a 134 bp distant CpG site [164]. The strongest association with HMW adiponectin was detected for rs2239857 (C>G) and rs77068073 (C>T), two rare variants flanking the CpG island, the lowest protein levels characterizing those heterozygous for both variants. The common variants rs12443878, rs12444338, rs62040565, and rs8060301 were found to affect the methylation level of up to three neighboring CpG sites. rs12444338 (G>T) and rs8060301 (T>A) were found to be associated with significantly lower adiponectin levels, and in strong LD with rs3865188 and rs4783244, situated at 9.7 kb and 455 bp upstream from the CpG island [32,81,164]. The strongest association with the level of methylation was detected between cg09415485 and rs8060301, influencing HMW adiponectin levels, HDL cholesterol concentration and diastolic blood pressure [164].

## 8. Insight to Complex Interactions Between Genetic and Non-Genetic Factors Influencing Adiponectin Levels

Although gene–gene and gene–environment interactions remain largely uninvestigated, existing observations suggest a highly complex background of circulating adiponectin levels as a multifactorial trait.

A limited number of studies aimed to clarify the role of genetic variation in molecules involved in the regulation of adiponectin expression. Genetic variation in transcription factors responsible for the transcriptional regulation or miRNAs involved in the post-transcriptional regulation of the adiponectin system may influence adiponectin levels indirectly. A systematic analysis of the polymorphisms of the main transcription factors involved in the control of *ADIPOQ* expression found a significant association only in the case of the *SREBF1* variant rs1889018, explaining approximately 0.4% of adiponectin variation [167]. In total, 232 monozygotic twin pairs were investigated to identify environmental effects on adiponectin levels mediated by DNA methylation, and to elucidate the genetic factors determining a different sensitivity to these changes [168]. GWAS of discordant and concordant twin pairs suggested that *CDH13* variation might determine the genetic effect on within-pair differences in adiponectin levels by influencing the sensitivity to epigenetic changes [168].

The inconclusive findings of association studies may be related to the significant differences among the various study populations regarding ethnicity, sex, age, life stage and environmental factors, especially diet. Racial differences related to genetic and environmental factors may be critical, as demonstrated by the often population-specific findings of plasma adiponectin association studies not replicated in samples of different ancestry. Differences in men and women were also reported [124,169,170]. For example, gender-dependent association was found with adiponectin levels in women for rs5444174, rs1681205, rs1403697 and rs7641507, consistent with haplotype analysis, in the comprehensive analysis of *ADIPOQ* variants in a large African American population from the Jackson Heart Study cohort [170].

Age could be an important factor rarely addressed. Critical life stages may affect the importance of variant effects on plasma adiponectin. For example, the *ADIPOQ* rs6773957 variant effect was found to be stronger during post-puberty [171]. The Hyperglycemia and Adverse Pregnancy Outcome Study investigated how metabolic changes induced by pregnancy may enhance adipose tissue dysfunction in genetically predisposed women [103]. While rs900400 showed only nominal association with adiponectin levels in non-pregnant women, risk allele carriers demonstrated a lack of adipose tissue flexibility. T allele carriers manifested a stronger physiologic response of pregnancy-related hypoadiponectinemia, a potential adaptive mechanism to deliver more nutrients to the fetus [103].

Dietary differences are difficult to address due to the significant complexity, though they might offer explanation for some of the conflicting results. Moreover, nutrigenetic findings might contribute to more efficient personalized prophylactic and therapeutic recommendations. Few studies investigated the interaction of genetic variation and dietary factors modulating the plasma adiponectin phenotype [100,159,172,173,174,175,176,177]. Research in a Spanish sample found that the minor allele of rs2917570 was associated with higher adiponectin concentrations when adherence to the Mediterranean diet was low, and lower adiponectin concentrations when adherence to the Mediterranean diet was high [100]. In the case of rs1501299, improvement of circulating adiponectin and metabolic parameters after hypocaloric dietary interventions could be demonstrated only in non-T-allele carriers, depending on the type of diet [172,175,176,177]. After a 12-week weight loss intervention in obese individuals, the significant decrease in insulin resistance and increase in adiponectin levels were observed in non-carriers of the T-allele [172]. After 9 months of hypocaloric dietary interventions in obese patients, increasing adiponectin levels were noted only in the absence of the T allele, and the obese individuals with the T allele showed no improvement of circulating adiponectin despite the weight loss [177]. In response to massive weight loss by biliopancreatic diversion surgery in morbidly obese individuals, the GG genotype was associated with increased adiponectin levels and better metabolic improvement [178].

## 9. Current Challenges and Perspectives of Adiponectin Genetics Research

Circulating adiponectin levels represent an intermediate phenotype important for health and disease, and a quantitative trait with polygenic inheritance that is still incompletely clarified. The first thirty years of adiponectin genetics research contributed significantly to our understanding of the physiological roles and involvement in pathology of this unique cytokine. Furthermore, genomics constitutes one of the most promising strategies to explain current knowledge gaps and apparent contradictions and it may help clarify causality in various pathologies. Genetics may also aid evaluating the importance of the adiponectin system in the clinical setting. Genomics may be applied to elucidate its utility as a diagnostic or prognostic biomarker and to capitalize on its therapeutic potential.

A crucial element of research is adequate phenotype assessment. Some of the conflicting results and paradoxes may be related to analytical limitations. Research on adiponectin genetics demonstrated the importance of the choice of analytical methods to avoid inaccurate quantification and isoform assessment that may lead to contradictory results [144]. Plasma adiponectin investigations in rare genetic variants offered unique information about the biological relevance and measurement challenges of the various isoforms [144,179].

Circulating adiponectin levels are considered remarkably stable despite a high level of production and rapid clearance [180]. Both production and clearance may be influenced by genetic factors in various ways, and while there are abundant data about adiponectin production, its clearance remains less investigated.

Classical family studies demonstrated increased heritability. However, the estimated values are highly variable, which may be related to the significant differences in study designs and population characteristics. Moreover, some observations suggest that environmental effects could be more important than in the case of similar adipokines or related traits [51]. Overall, the value of heritability estimates remains controversial, and the information offered is limited. Heritability values are valid for the population and particular environment investigated, and reveal no data about the specific contributing factors.

According to genome-wide linkage and association studies, numerous candidate genes located on almost all chromosomes could be involved. GWAS proved successful in identifying a high number of common genetic variants associated with circulating adiponectin levels and related metabolic traits. However, the results are often inconclusive, and sometimes contradictory. The mechanisms of action usually remain unclear, and the minor effects fail to explain the heritability estimates. The missing heritability could be related to rare variants, other than single nucleotide variation, epigenetic mechanisms, gene–gene and gene–environment interactions, which remain largely uninvestigated.

Association of a locus with circulating adiponectin does not demonstrate that the implicated gene directly regulates adiponectin. Positive association may mean causality, but also LD with a causal variant located in the region or an admixture artifact. LD is suspected in the case of several important *ADIPOQ* and *CDH13* variants. Highly conflicting association study results are often related to the mixed nature of the investigated populations, and could be clarified by the use of internal control genotypes and relatively homogeneous populations. Inclusion of underrepresented demographics, rare and other than single nucleotide variants will be necessary.

Adiponectin genetics research introduced an efficient and cost-effective strategy with potential for generalization for the identification of rare variants with large effects. High-impact population-specific variants were identified using a combination of linkage analysis, whole exome and direct sequencing, and association methods in a family-based approach [108]. The increasing access to next-generation sequencing may assist the identification of rare variants, while long-read sequencing may detect complex structural variations.

Advanced statistical methods may allow multilocus and interaction testing. GWAS meta-analyses, data from combined database cohorts may offer increased statistical power, precision and validity in case of minor effects, and could clarify hiding heritability. Machine learning using large genetic, demographic and phenotype datasets may assist addressing at a new level subtle effects and complex interactions between genetic and non-genetic factors modulating adiponectin levels.

Functional analysis of the variants is limited. Classical reductionist approaches like knock-in or knock-out animal studies are inadequate to address inter-specific gaps and the characteristic complexity [144]. Investigation of genetic variants by novel in silico methods or gene editing may improve our understanding of adiponectin biology and involvement in pathology.

Long-read RNA-sequencing may improve transcript quantification and offer insight into alternative splicing. High throughput experimental and computational methods and integrated omics approaches may clarify gene expression regulation and variant effects. Post-translational modifications are major contributors to the non-linear relationship between mRNA and protein levels, and the mutations directly or indirectly affecting oligomer assembly and multimerization are particularly important for adiponectin secretion, blood levels, tissue accumulation and specific isoform effects.

Innovative spatio-temporal omics technologies may clarify specific cellular and dynamic changes in intermediate phenotypes related to genetic variation, interactions and clinical phenotypes. Phenome-wide association studies may also clarify the pleiotropic effects of certain variants. By quantifying and integrating intermediate phenotypes like mRNA and protein levels, systems genetics may address characteristic complexity. High-throughput omics technologies may clarify the effect of natural genetic variation on molecular phenotypes, physiological or pathological traits, as well as complex interactions, including dietary responses that remain largely uninvestigated.

Circulating adiponectin levels act as a mediator crucial for explaining the association between genetic variants and complex traits. Association of genetic variants with altered adiponectin levels and downstream diseases, however, does not necessarily establish a causal effect. Elaborate phenotyping of rare mutations associated with significantly reduced plasma levels but not altered metabolism has put into question the role of adiponectin especially as an insulin-sensitizer [108]. Correlation between molecular and clinical traits may mean causality but also independent phenotypes modulated by a confounding factor. Causality may mean that circulating adiponectin is causal or reactive to diverse physiological or pathological phenotypes. Linkage studies suggested shared genetic architectures. Co-mapping to 3q27 provided evidence for the relationship between circulating adiponectin levels and metabolic traits and diseases, suggesting causality. Mendelian randomization is useful to study causality overcoming confounding and reverse causation. So far, Mendelian randomization analyses yielded conflicting results in diverse pathologies, but this strategy could become more informative by adequate phenotyping and comprehensive variant characterization.

Moreover, research on the genetic background of circulating adiponectin levels may help to not only decipher fundamental biological mechanisms but also the development of novel therapeutic and diagnostic strategies. Studies addressing the complex gene–gene and gene–environment interactions could be important for developing predictive, preventive, personalized genomic medicine approaches, allowing better risk assessment by polygenic risk scores, or more efficient recommendations based on nutrigenetic and pharmacogenetic implications [150,172,173,174,175,176,177]. *ADIPOQ* is also considered a longevity gene, and adiponectin might represent a key biomolecule at the confluence of adiposity and aging [161,181]. Novel genomic research may reveal information of practical importance about adiponectin as a modulator of healthspan and lifespan, and could clarify the impact of therapeutic targeting of the adiponectin system on morbidity and mortality.

## 10. Conclusions

Circulating adiponectin levels represent an intermediate phenotype critical for understanding adiponectin biology and medical importance. It is a complex quantitative trait determined by a multifactorial background in which heritability estimates based on classical family studies, hypothesis-free genome-wide linkage scans and association studies demonstrated the importance of genetic factors.

Tens of loci on almost all chromosomes and numerous variants in over 150 genes might be involved in modulating the phenotype, and complex interactions may exist between genetic and non-genetic internal and external factors. The most important determinants are *ADIPOQ* and *CDH13*, characterized by genetic variation with significant population differences. While in populations of European ancestry, genetic variation in the gene encoding adiponectin is the dominant determinant of plasma adiponectin variance, in populations of East Asian ancestry, polymorphisms of the gene encoding T-cadherin appear particularly important. High-impact population-specific rare missense adiponectin mutations cause hypoadiponectinemia by altered trimer or multimer assembly, while common low-impact non-coding or synonymous variants, often in linkage disequilibrium, may associate with adiponectin deficiency due to perturbed gene expression regulation.

Improved phenotyping, as well as emerging omics and computational technologies, could address and clarify the complex multifactorial background of circulating adiponectin levels, the conflicting observations and knowledge gaps of the first three decades of adiponectin genetics research.

## Figures and Tables

**Table 2:** Quantitative trait loci (QTLs) mapped for serum adiponectin levels [69].

QTL	Phenotype (MIM Number)	Cytogenetic Locus	Genomic Coordinates (GRCh38)	Reference
ADIPQTL1	#612556	3q27.3	3:186,842,710–186,858,463	[26]
ADIPQTL2	%606770	5p15.2-p14	5:9,900,001–28,900,000	[52]
ADIPQTL3	%606771	14p11.2-q13	14:8,000,001–37,400,000	[52]
ADIPQTL4	%612629	11q23-q24	11:110,600,001–130,900,000	[70]
ADIPQTL5	%613836	16q23.3	16:81,600,001–84,100,000	[32]

## Data Availability

No new data were created or analyzed in this study. Data sharing is not applicable to this article.
